# Supplementary material for: An open-source platform for head-fixed operant and consummatory behavior
Source: eLife. 2023 Aug 9;12:e86183. doi: 10.7554/eLife.86183 (PMC10499376; doi:10.7554/eLife.86183)
Supplement: Supplementary file 1. — Detailed protocol for OHRBETS (Open-Source Head-fixed Rodent Behavioral Experimental Training System) assembly. [file elife-86183-supp1.pdf]

System Overview

Behavioral Stage

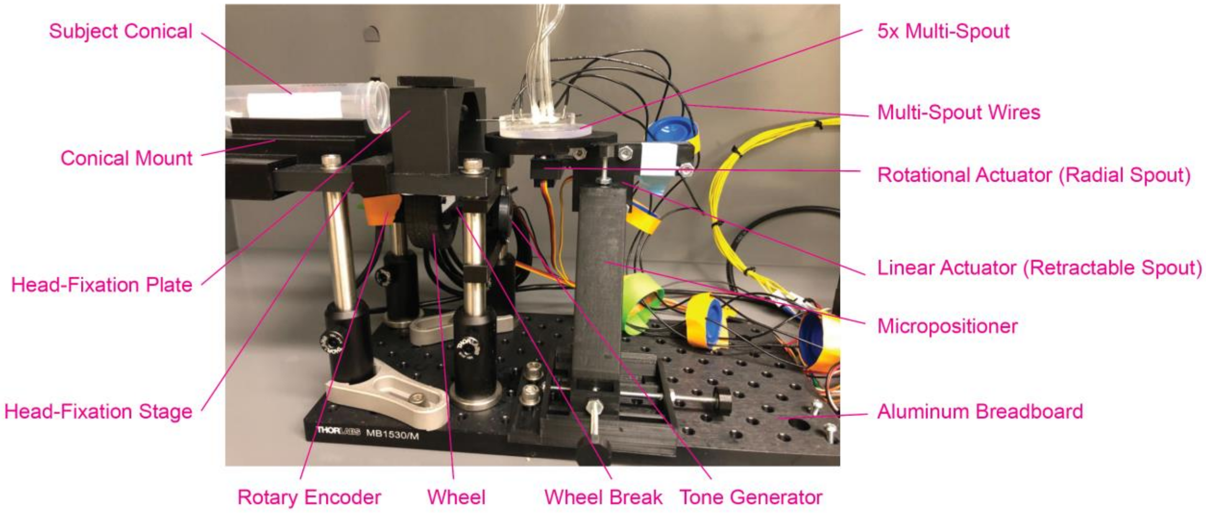

Behavioral Box Internal

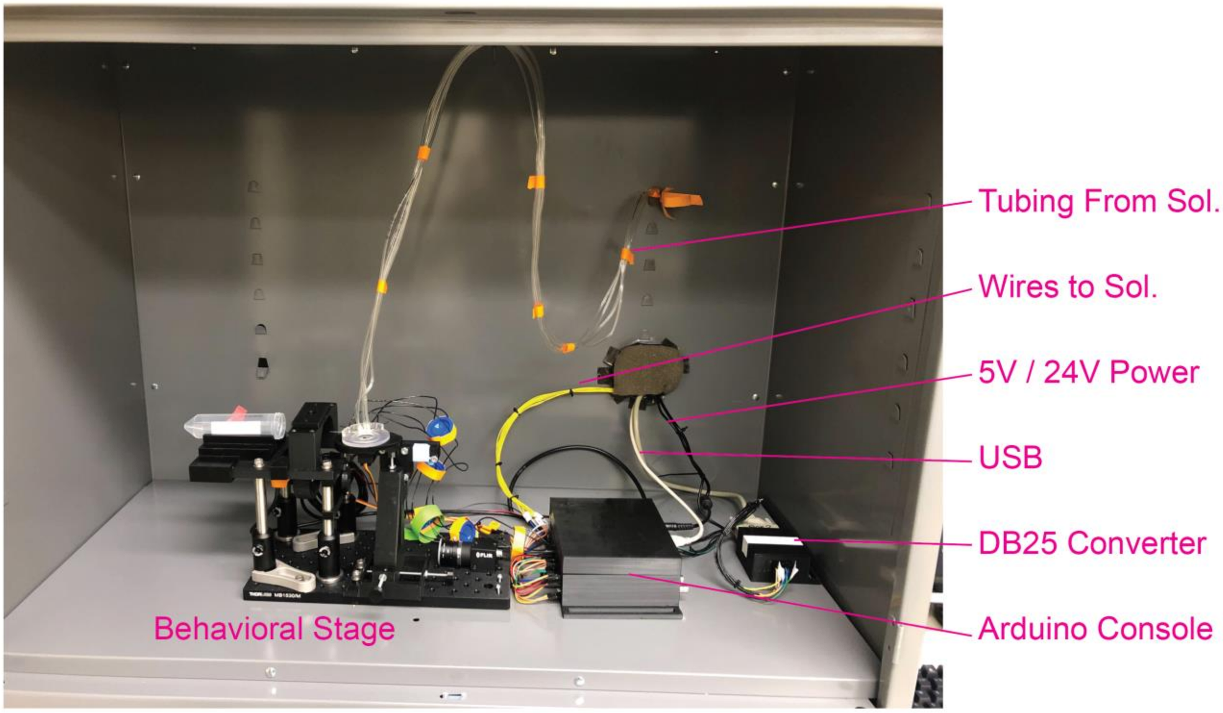

Behavioral Box External

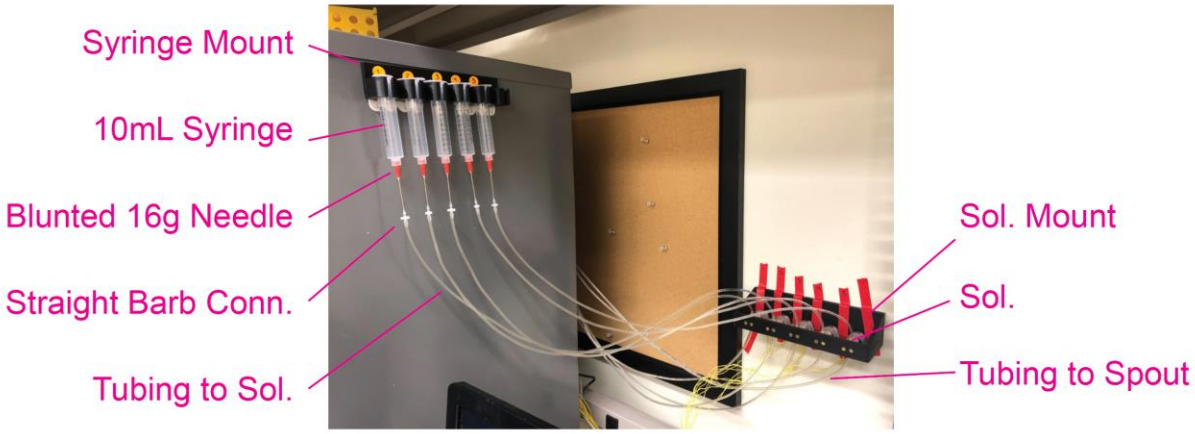

Arduino Console Internal Connectivity Key

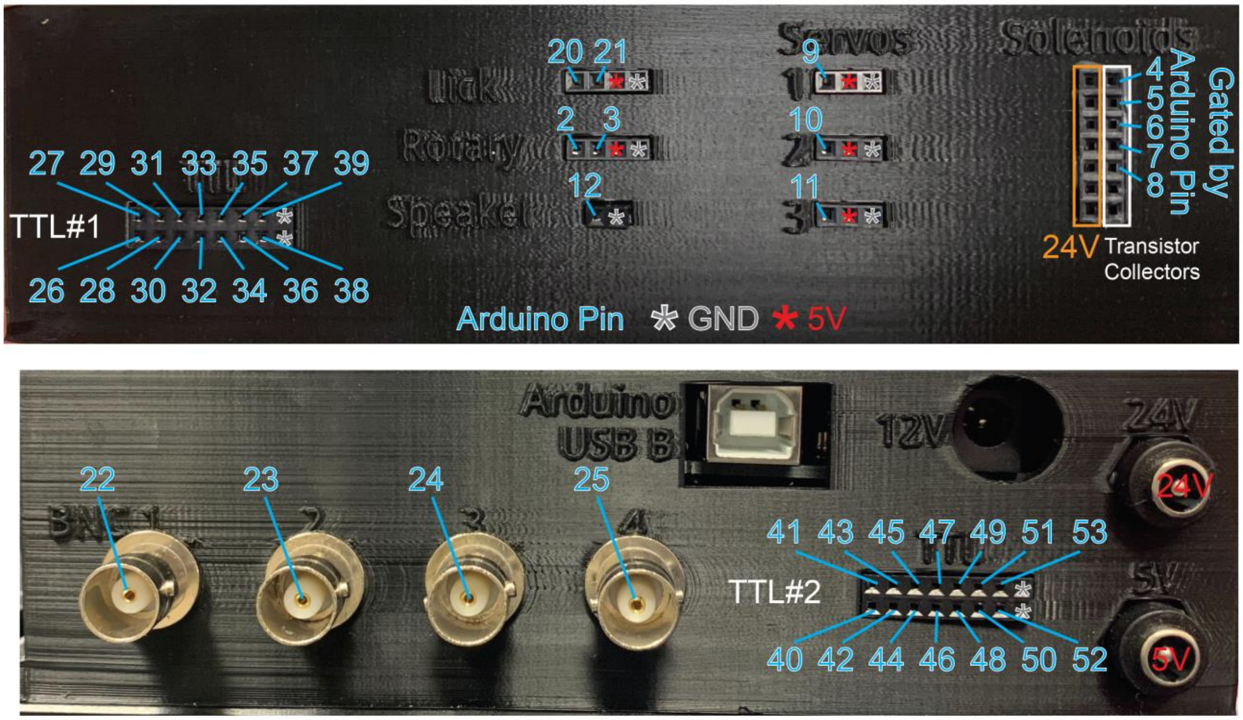

Arduino Console External Connectivity Key

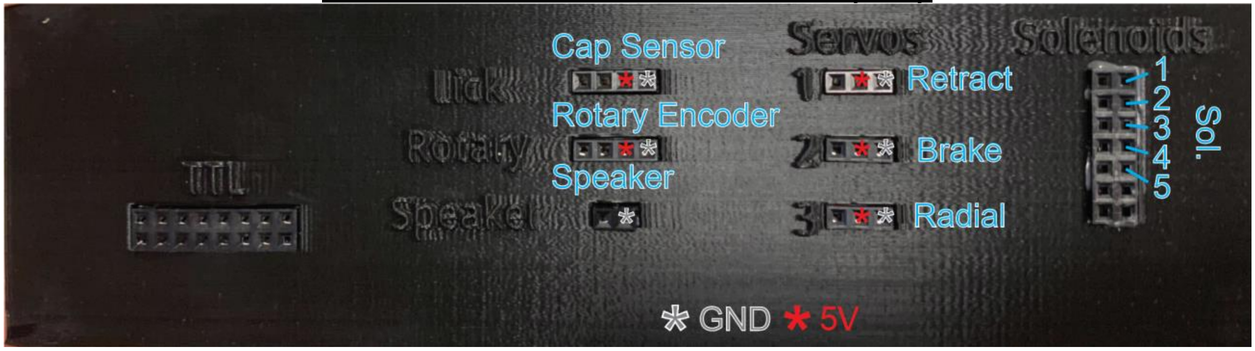

Hardware

This section consists of descriptions of the main components of the head-fixed system and is broken into two subsections: 1) 3D printed components and 2) Electrical components. This section should serve as a reference to help with identifying components and understanding relations between components. The electrical component section will also serve as a quick guide to proper wiring.

Refer to purchase_list.xlsx for full list of components.

In addition to the hardware outlined here, you will need to have head-rings machined from stainless steel (see *headring.stl* for dimensions). If you plan to use the system for two-photon imaging, you will need to have the ***Head-Fixation Plate*** and ***Head-Fixation Top Piece*** machined from stainless steel as well (ideally, the ***Head-Fixation Plate*** will be tapped for M3 screws).

3D Printed Components

This subsection contains all the 3D printed components used for the behavioral system. Unless otherwise noted, each piece can be printed using filament. Each section includes images from one or more of the following open-source Tinkercad files:

- Assembly (link)
- Components Filament (link)
- Components Resin (link)
- Components Multi-Spout (link)

All components are also available as .stl files in the GitHub 3D_models subfolder.

Head-Fixation Stage:

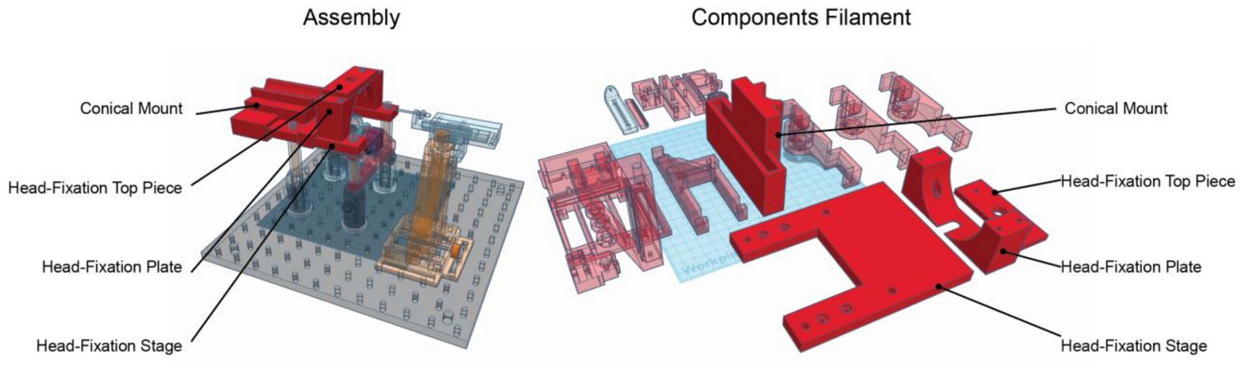

Purpose:
- To stably mount subjects with head-ring implantations.

Components:
- ***Stage***: Stage that is fixed to optical posts and mounted to aluminum breadboard and serves as a platform additional components are mounted to.
- ***Head-Fixation Plate***: Horizontal plate with a head-ring that the subject's head-ring is slotted on top of during head-fixation.
  - *Note: We advise having back-ups of this component because with extensive use this piece can be worn down to the point that the head-rings slide through the plate*.
- ***Head-Fixation Top Piece***: Plate that is tightened downward to hold subject's head-ring in place.
  - *Note: Particular applications require versions of this top piece exist for particular applications (e.g. accommodating the Inscopix baseplate).*
- ***Conical Mount***: Removable adapter for mounting subject conical. This component can be swapped out for different applications (larger subjects, tail fixation, etc.)

Wheel Mount

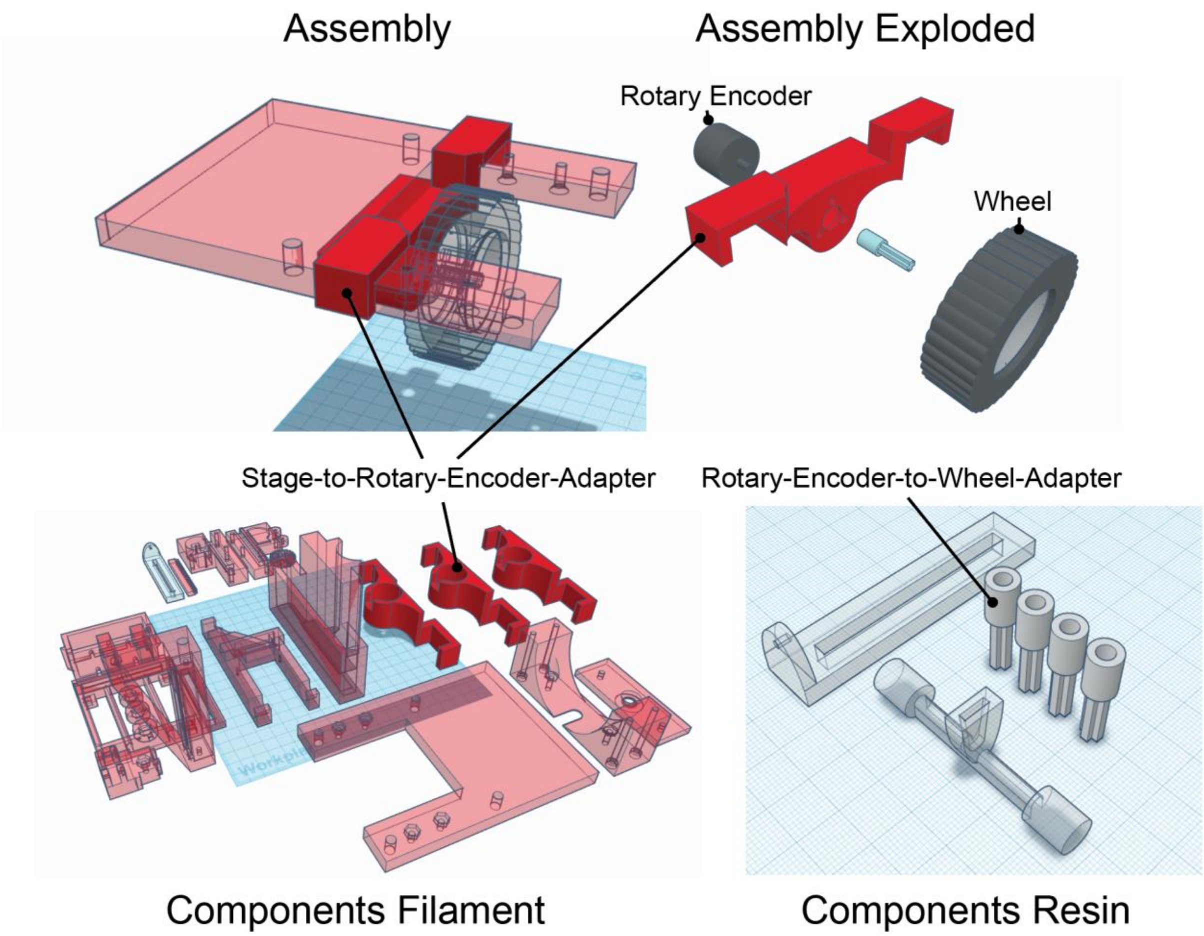

Purpose:
- To mount the ***Rotary Encoder*** and wheel directly beneath the head-fixation stage.

Components:
- ***Stage-to-Rotary-Encoder-Adapter***: Attaches ***Rotary Encoder*** to the ***Head-Fixation Stage***.
    - Options: 3 different heights are included for different approaches (subjects with different sizes or head-rings of different heights). Default is the mid-height option.
- ***Rotary-Encoder-to-Wheel-Adapter*** (*resin*): attaches the ***Wheel*** to the ***Rotary Encoder***
    - 3D model includes multiple replications of the pieces with slightly different inner diameters. The component should fit very snugly on the rotary encoder and small imperfections from different prints can result in different inner diameters fitting best. After 3D printing is complete, test the diameter of each adapter and discard ones that don't fit snug.
- ***Wheel***: Lego Wheel 43.2mm D. x 18mm - Flush Axle Stem with Black Tire 62.4 x 20 S (86652 / 32019) (see www.bricklink.com/, or an imitation piece see link)

- ***Rotary Encoder*** (see *Electrical Components- Rotary Encoder*)

Wheel Brake

Assembly Assembly Exploded

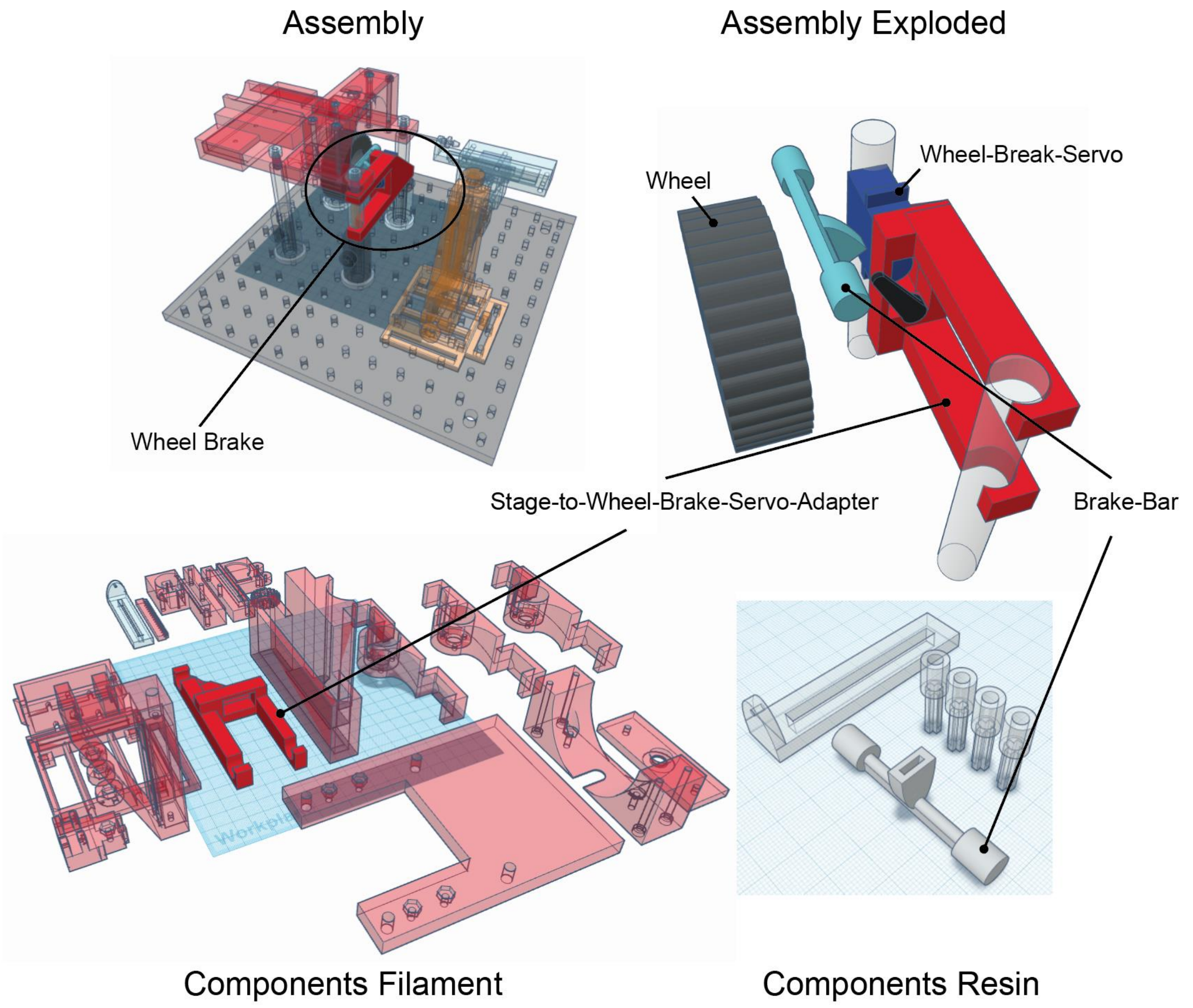

Filament Components Resin Components

Purpose:

- The wheel brake is used to prevent wheel rotation thereby limiting free access to operant responding.

Components:

- ***Stage-to-Wheel-Brake-Servo-Adapter***: Attaches the ***Servo*** that controls the ***Brake Bar*** to the optical posts of the ***Head-Fixation Stage***.
- ***Servo for Wheel Brake*** (see *Electrical Components- Servos*): Servo that is used to raise and lower the ***Brake Bar***
- ***Brake Bar*** (*resin*): Brakes the ***Wheel*** when lowered

Micropositioner
The ***Micropositioner*** design is adapted from Backyard Brains (https://backyardbrains.com/products/micromanipulator).

Assembly | Assembly Exploded

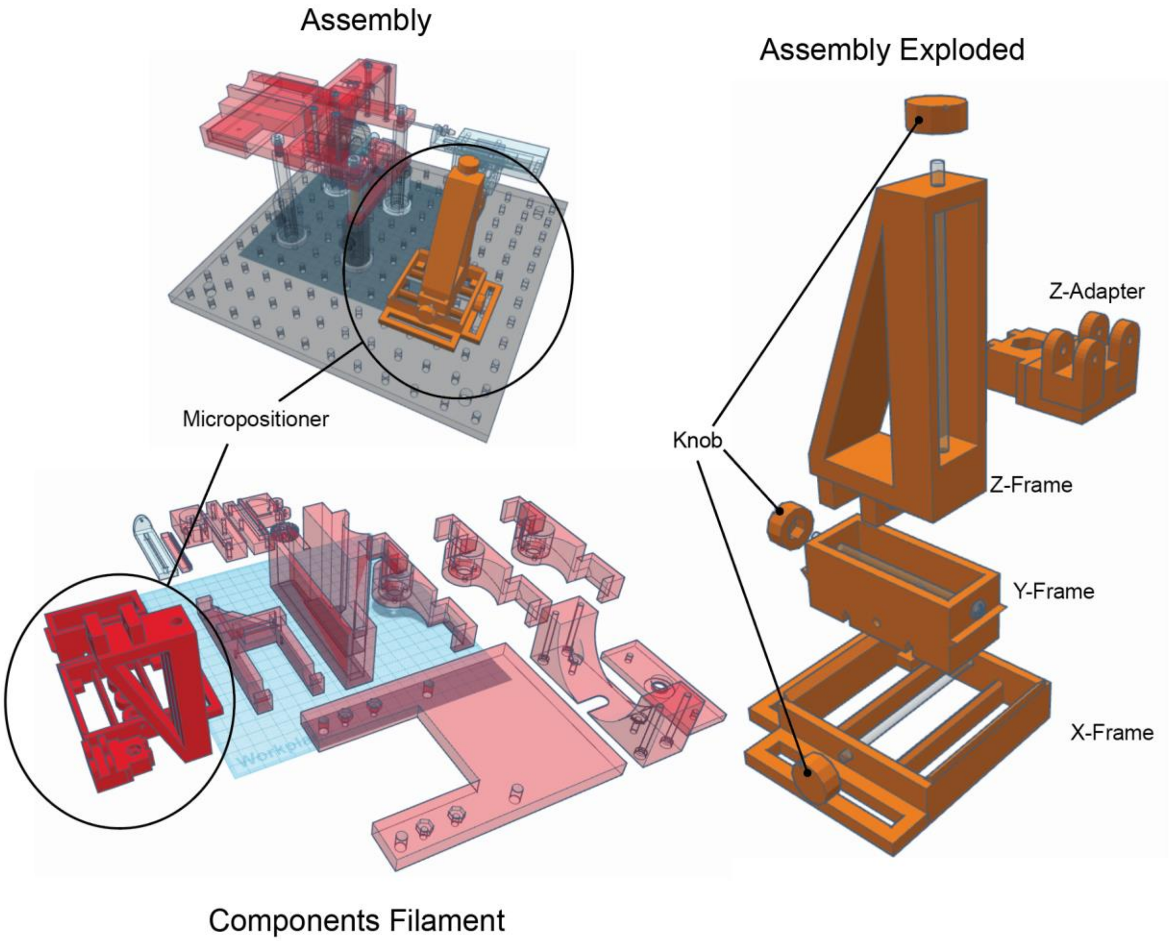

Purpose:
- The ***Micropositioner*** is used to position the ***Spout*** directly in front of the subject.

Components:
- ***X-Frame***: allows positioning in X dimension.
- ***Y-Frame***: allows positioning in Y dimension.
- ***Z-Frame***: allows positioning in Z dimension.
- ***Z-Adapter***: ***Micropositioner*** to ***Linear Actuator***.
- ***Knob*** (x3): used to rotate axels for positioning in each dimension.

Linear Actuator for Retractable Spout (Single Spout)

The ***Linear Actuator*** design is adapted from https://hackaday.io/project/10578-3D-printed-linear-servo-extender.

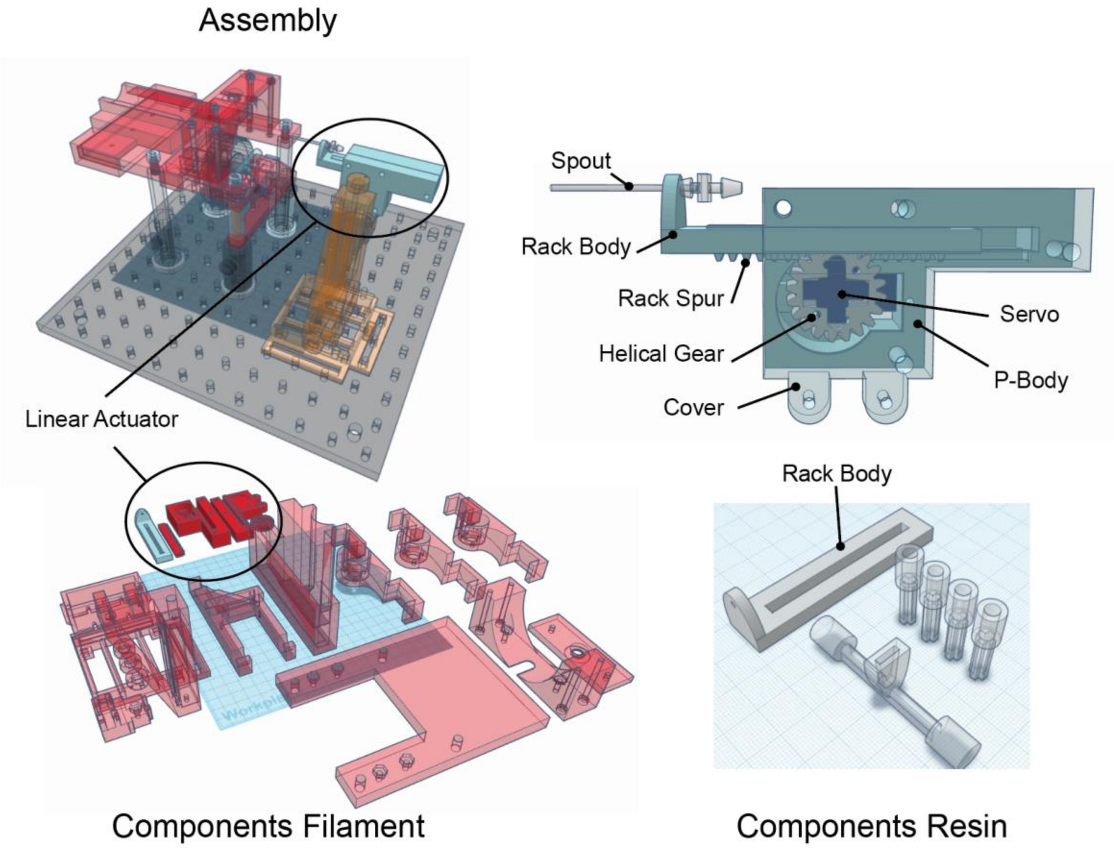

Purpose:

- The ***Linear Actuator*** allows the ***Spout*** to be retracted and extended, thereby regulating access to consumption.

Components:

- ***Spout*** (see below for building instructions): delivery of liquid to the subject.
- ***Rack Body*** (*filament or resin*): Mounts the ***Spout*** and seats the ***Rack Spur*** which allows the ***Rack Body*** to travel forward and backward.
  - The ***Rack Body*** can be made of filament or resin. Resin produces the best fit for the ***Spout***, but with the right settings filament can work as well.
- ***Rack Spur***: Rests inside ***Rack Body*** and interfaces with the ***Helical Gear*** and transfers rotation of ***Helical Gear*** to linear travel.
- ***Helical Gear***: Transfers ***Servo*** rotation to movement of ***Rack Spur***.
- ***P-Body***: Mounts ***Servo*** and holds ***Helical Gear***, ***Rack Body***, and ***Rack Spur*** in alignment.
- ***Cover*** (transparent in image): Works with ***P-Body*** to hold components in alignment and interfaces with ***Z-Adapter*** of the ***Micropositioner***.
- ***Servo for Linear Actuator*** (see *Electrical Components- Servos*)

Linear Actuator & Radial Actuator for Multi-Spout

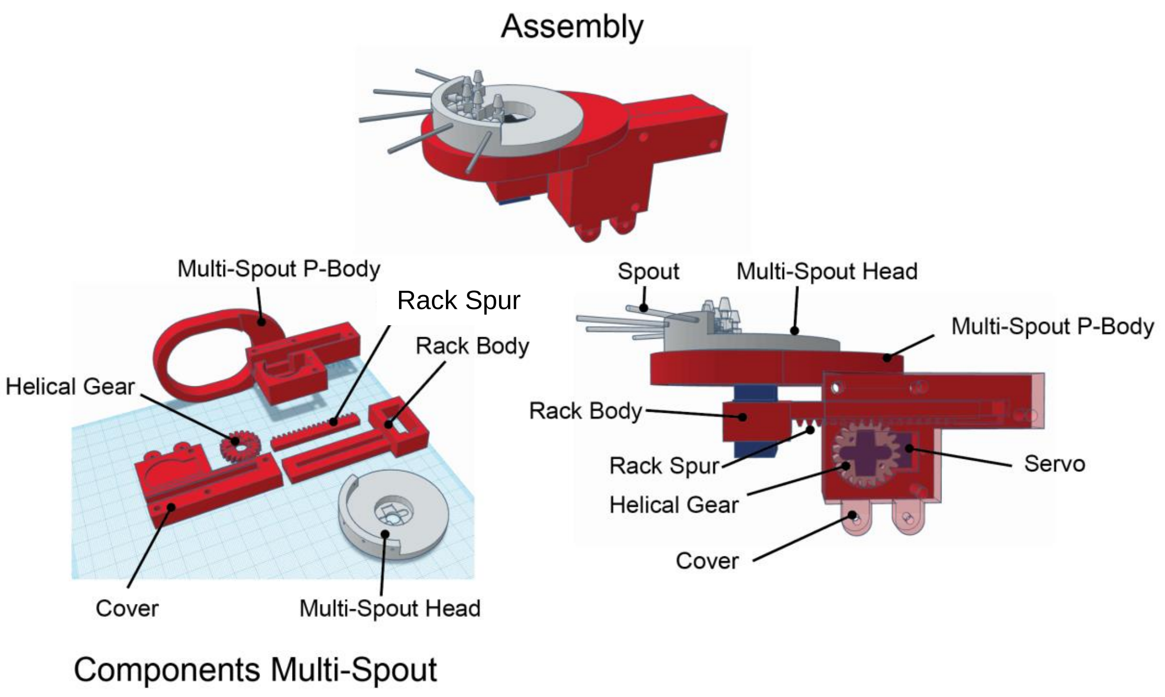

Purpose:

- The ***Linear Actuator*** allows the ***Spout*** to be retracted and extended, thereby regulating access to consumption. The radial actuator allows the ***Multi-Spout Head*** to be rotated to allow access to one of multiple ***Spouts***.

Components:

- ***Spout*** (see below for building instructions): delivery of liquid to the subject.
- ***Rack Body***: Mounts the ***Servo*** for radial travel and seats the ***Rack Spur*** which allows the ***Rack Body*** to travel forward and backward.
- ***Rack Spur***: Rests inside ***Rack Body*** and interfaces with the ***Helical Gear*** and transfers rotation of ***Helical Gear*** to linear travel.
- ***Helical Gear***: Transfers ***Servo*** rotation to movement of ***Rack Spur***.
- ***P-Body***: Mounts ***Servo*** and holds ***Helical Gear***, ***Rack Body***, and ***Rack Spur*** in alignment.
    - *Note: The multi-spout **P-Body** also has a support for the **Multi-Spout Head** that reduces the risk of damage to the **Multi-Spout Head**.*
- ***Cover*** (transparent in image): Works with ***P-Body*** to hold components in alignment and interfaces with ***Z-Adapter*** of the ***Micropositioner***.
- ***Multi-Spout Head***: Mounts multiple spouts angled relative to the center of the ***Multi-Spout Head***.
- ***Servo for Linear Actuator*** (see *Electrical Components- Servos*).
- ***Servo for Radial Actuator*** (see *Electrical Components- Servos*).

3D Printed Stage + Posts (optional)
This 3D model can replace the 3D printed stage and optical posts used in the system presented above (link).

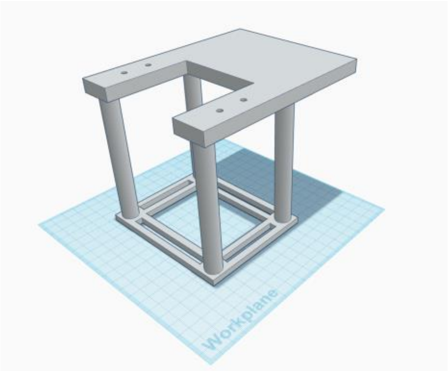

Note: The advantage of this model is that it reduces the cost of the system by replacing the need for optical posts, post mounts, and clamping forks. The disadvantage of the model is that it does not have adjustable height.

Solenoid Mounts
Mount for attaching ***Solenoids*** to side of box or nearby wall (link).

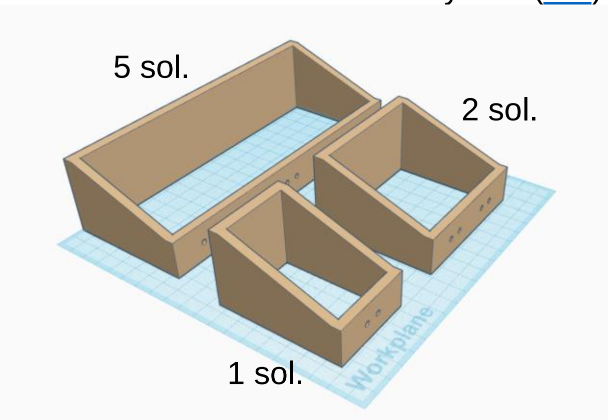

Syringe Mounts
Mount for attaching ***Syringes*** to side of box or nearby wall (link).

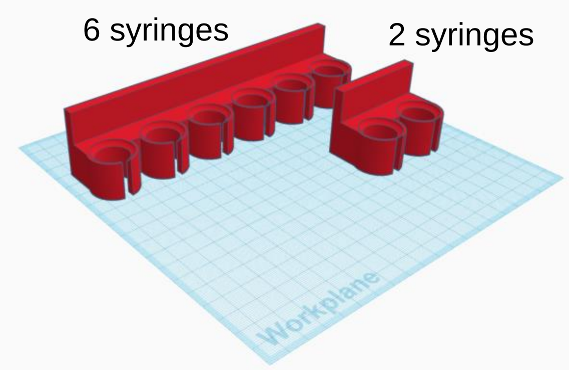

Electrical Components

Microprocessor

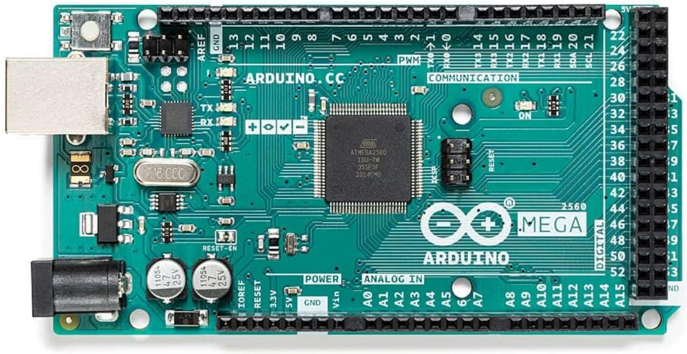

Purpose:

- To control behavioral hardware and to record behavioral events.

Component:

- Arduino Mega 2560 Rev 3

Note: Other Arduinos can be used in place of this model. However, the capacitive touch sensor has associated libraries that may not be compatible with all Arduinos.

Capacitive Touch Sensor (MPR121)

Purpose:

- To detect tongue contacts on each ***Spout***.

Component:

- Adafruit MPR121

Circuit:

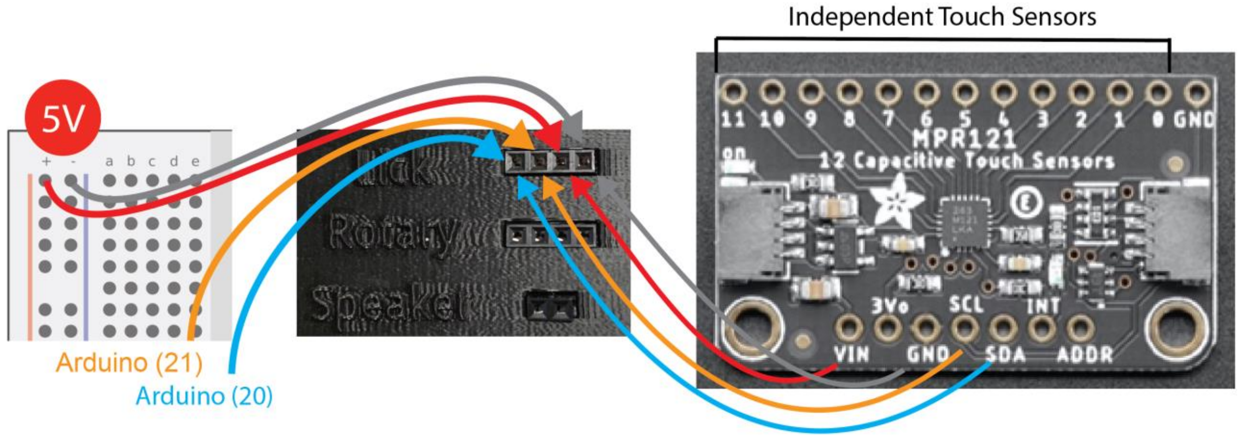

Connections:

- VIN → 5V input
- GND → Ground
- SDA → SDA on Arduino MEGA pin 20
- SCL → SCL on Arduino MEGA pin 21
- 1-n → ***Spout(s)*** 1-n

Solenoid and Solenoid Circuit

Purpose:

- The ***Solenoid*** and ***Solenoid*** circuit allows the Arduino output pins with 5V to open the ***Solenoid*** using 24V thereby allowing liquid of set volumes to flow.

Components:

- ***Solenoid*** (Parker Series 3 – Miniature)
    - *Note: leads on the solenoid are non-polar (either can be used for + or -)*
- ***Transistor*** (Tip 120)
- ***Resistor*** (5.6kOhm)
- ***24V Power Supply***

*Note: For each individual **Solenoid**, there must be an independent **Solenoid Circuit**.*

Circuit:

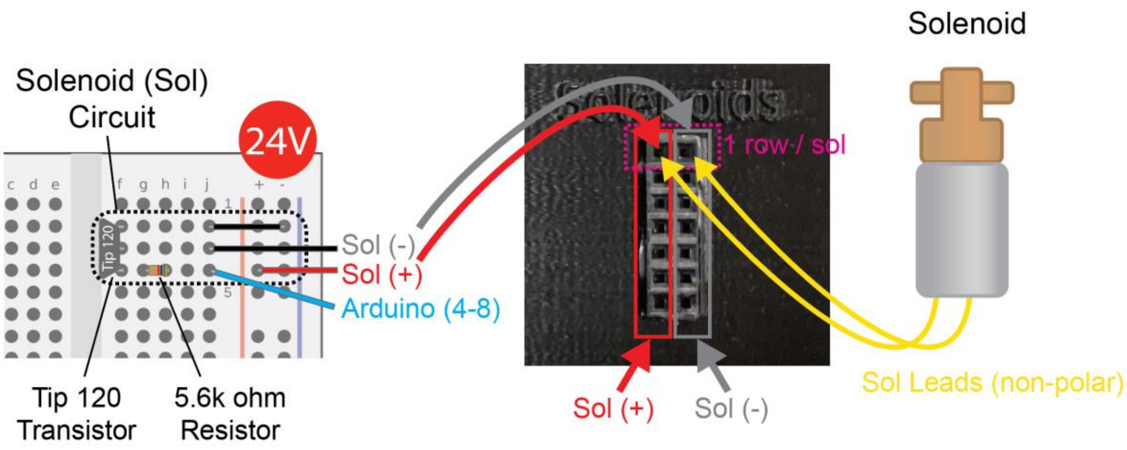

Connections:

- ***Solenoid*** leads
    - 24V (+)
    - Transistor Collector (middle row in solenoid circuit above)
- ***Solenoid Circuit*** (1 per solenoid)
    - Base: Wire from Arduino pin + 5.6kOhm resistor in parallel
    - Collector: ***Solenoid*** lead
    - Emitter: Common ground

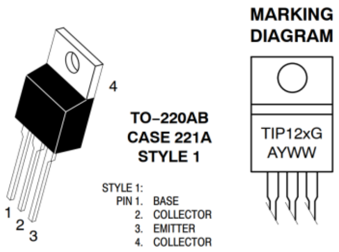

Source: https://www.onsemi.com/pdf/datasheet/tip120-d.pdf

Servos

Purpose:

- Each ***Servo*** controls specific components of the system. The absolute rotational position of each ***Servo*** can be set independently using unique Arduino pins. There are up to 3 ***Servos***, depending on the system configuration:
  - ***Wheel Brake***
  - ***Linear Actuator*** for retractable ***Spout***
  - ***Radial Actuator*** for ***Multi-Spout***

Component:

- [Tower Pro SG92R]

Circuit:

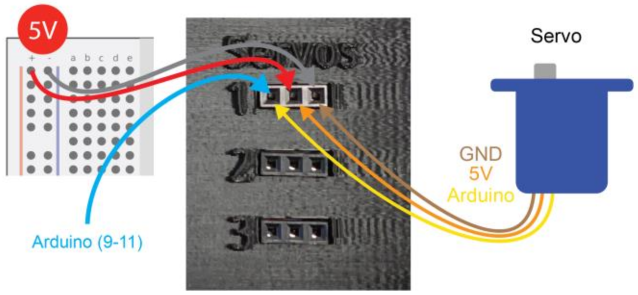

Connections:

- Ground (Brown on ***Servo***) → Common ground
- 5V Power (Orange on ***Servo***) → 5V
- Signal (Yellow on ***Servo***) → Arduino pin

Rotary Encoder

Purpose:

- To measure wheel rotation. The ***Rotary Encoder*** used in this system is an optical, incremental encoder that measures *relative rotation.*

Component:

- Kubler Incremental Encoder 1024 ppr, 05.2400.1122.1024

Circuit:

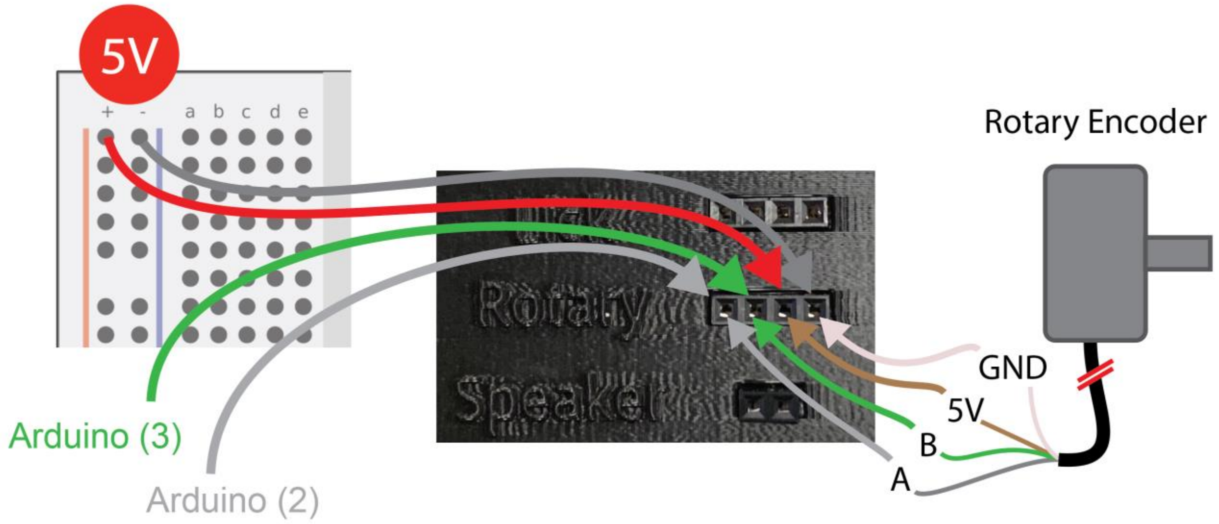

Connections:

- Ground (White) → Common ground
- 5V Power (Brown) → 5V power supply
- Rotary encoder A (Grey) → Arduino pin (2)
- Rotary encoder B (Green) → Arduino pin (3)

Note: The Arduino script reading the rotary encoder relies on interrupt service routines (ISR) which are only possible on specific Arduino pins. It is critical that the rotary encoder A and B are attached to these specific pins. Our scripts are written to have rotary encoder A on pin 2 and B on pin 3.

Assembly Instructions

In this section, we will first assemble the following subcomponents:

1) ***Micropositioner***
2) ***Lick spouts***
3) ***Linear Actuator (single spout)***
4) ***Linear Actuator & radial actuator (multi spout)***
5) ***Rotary encoder***
6) ***Tone Generator***
7) ***Preparation of external electrical components***
8) ***Arduino circuit***

Then, we will assemble subcomponents into the final system.

See the Hardware section for a detailed guide to each of the 3D models and electrical components that will be used in this section.

Assembly of Subcomponents

Micropositioner

Components and building materials:

- 3D printed components
  - ***X-Frame***
  - ***Y-Frame***
  - ***Z-Frame***
  - ***Z-Adapter*** (Connects ***Micropositioner*** to ***Linear Actuator***)
  - ***Knob*** (3x)
- Other components
  - Machine screws (x3)
  - Hex nuts (x6)
- Tools and Materials
  - Vice clamp
  - Screwdriver
  - Super glue
  - JB weld epoxy (optional)
  - Magnets 1/8” (optional)

Follow the building instructions provided here with the following modifications:

- The angled ***Z-Frame*** in the original design has been replaced with a perpendicular one.
- the ***Z-Adapter*** has been replaced with one that allows for a more secure grip to the ***Cover*** of the ***Linear Actuator***.
- The ***X-Frame*** has been modified to include a rail to assist with mounting to the aluminum breadboard.

OPTIONS: If you are using a metal plate or the floor of your behavioral box as a stage instead of an aluminum breadboard, follow the steps in the instructions for attaching magnets under the ***Micropositioner***.

- These magnets will be sufficient for fixing the ***Micropositioner*** in place but will not be as robust as fixing to an aluminum breadboard.

TROUBLE SHOOTING:

- If the nuts disconnect from the machine screws during operation, then use JB weld in place of super glue for greater security.
- If the hex nuts do not fit into a ***Knob***, use a vice clamp to force them in place.

Spout

Components and building materials:

- Protective eyewear
- 16g needle (1 needle per ***Spout***)
- Dremel
- Cutting disk for Dremel (EZ456B)
- Vice clamp or other Dremel mount
- Plyers (or hemostat)

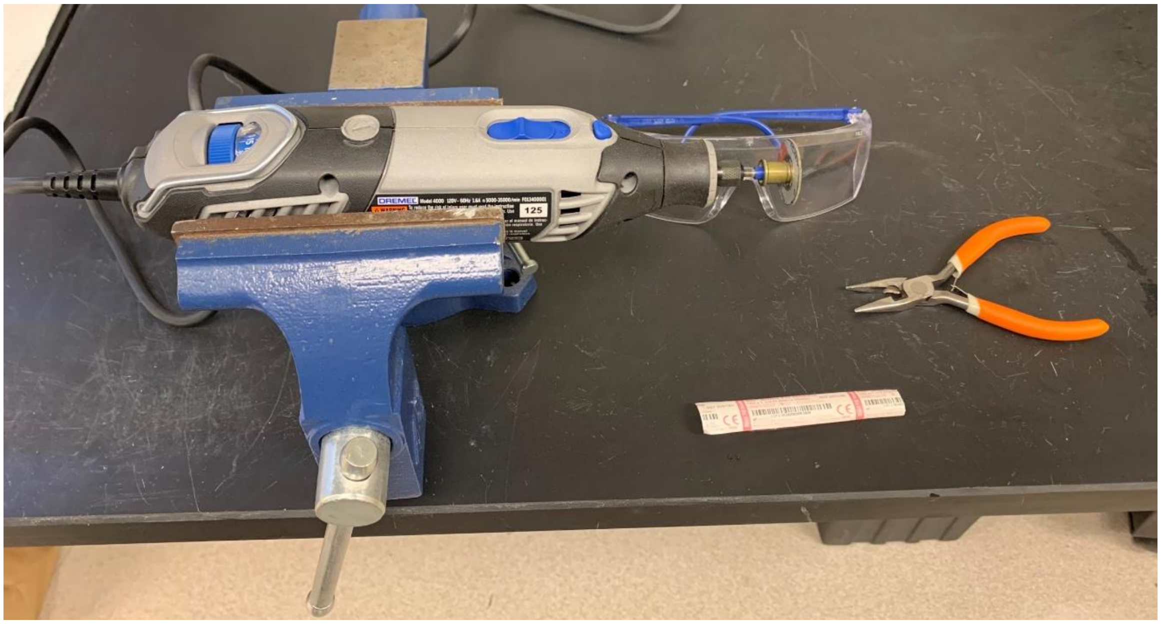

Instructions:

1) Put on protective eyewear.
2) Mount the Dremel in the vice clamp such that the Dremel is horizontal (see photo above).
3) Cut the 16g needle or hypodermic tubing
    a. Using a pair of plyers, hold the needle near the hilt leaving a small piece of metal exposed before the hub of the needle.
    b. Turn the Dremel on high.

c. Carefully press the exposed portion of the needle down onto the cutting disk until you can cut the needle in two.

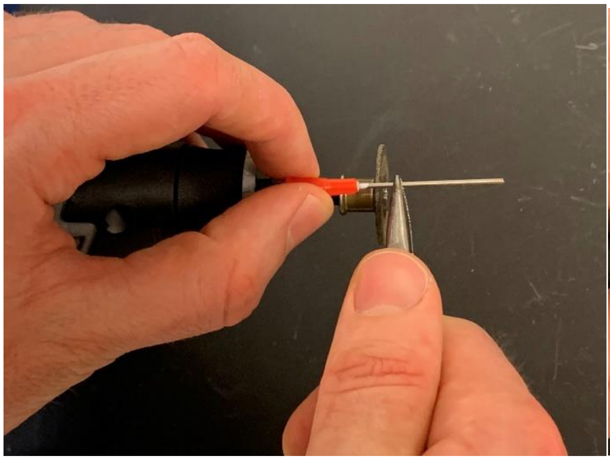
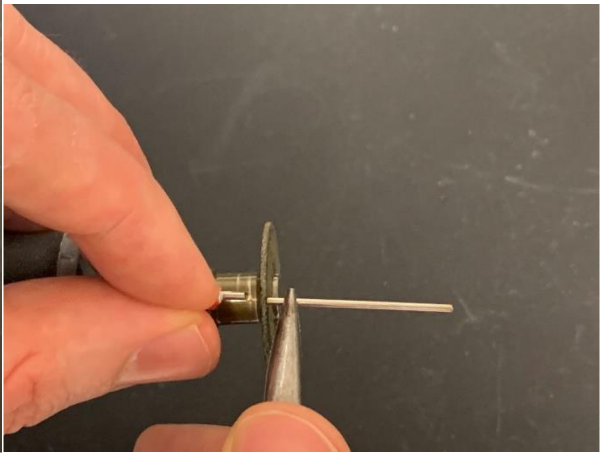

4) Smooth both ends of the needle
   a. Using a pair of plyers or your hand, hold the needle and gently press the bevel into the face of the cutting disc until you have completely sanded down the bevel. You should now have a dulled piece of tubing.

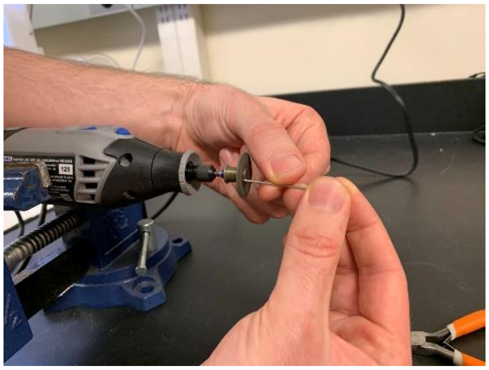

   b. Using your hands, hold the dulled tubing at an angle relative to the cutting disc. Gently press the tubing against the cutting disc and slowly rotate the tubing as you increase the angle to reach perpendicular with the sanding disk.

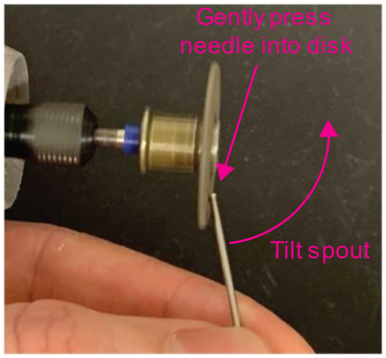

   c. Use your finger to feel how smooth the edges of the tubing are. If the tubing still has rough edges that snag on your skin or gloves, repeat the previous step until the edges are completely smooth.

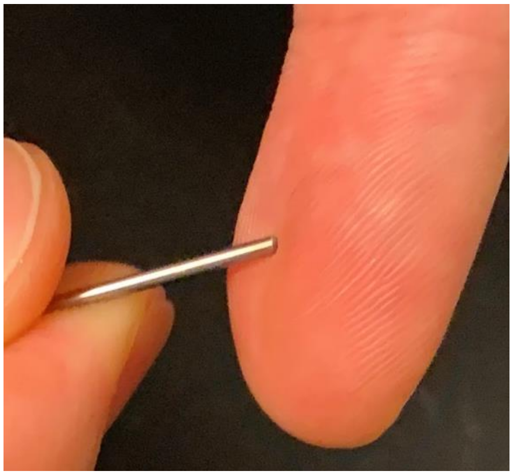

d. Use a length of stripped 23AWG solid core wire to widen the opening of the tip of the metal tubing and repeat step c to ensure that the tip has remained smooth.
e. Repeat the previous steps with the other side of the needle.

Multi-Spout Head

Components and building materials:

- 3D printed components
  - ***Multi-Spout Head***
  - ***Multi-Spout Head Assembly Guide***
- Prepared components
  - ***Spout*** (5x) (see above)
- Tools and materials
  - Vice clamp
  - Dremel with cutting disc
  - 23AWG *stranded* wire (not solid core)
  - FF jumper wires (5x)
  - Small diameter heat shrink tubing (1/8")
  - Wire stripper
  - Helping hands
  - Soldering iron
  - Solder
  - Angled barb connector (5x)
  - Super glue

Instructions:

First, we will create a ***Spout Assembly*** for each spout in the system that consist of a ***Spout*** soldered to a wire and attached to an angled barb connector. We will then insert each Spout Assembly into the ***Multi-Spout Head*** using the ***Multi-Spout Head Assembly Guide*** to hold each ***Spout Assembly*** in position. Finally, we will use super glue to fix each ***Spout Assembly*** to the ***Multi-Spout Head*** and remove the ***Multi-Spout Head*** from the ***Multi-Spout Head Assembly Guide***.

1) Solder each wire to a female jumper wire connector
    a. Cut a jumper wire with a female lead leaving ~10cm. Strip the jumper wire leaving ~1cm of wire exposed and twist wire.
    b. Cut a length of ~10cm length of 23AWG *stranded* wire. Strip one end to leave ~1cm exposed, and other end to leave ~2cm exposed.

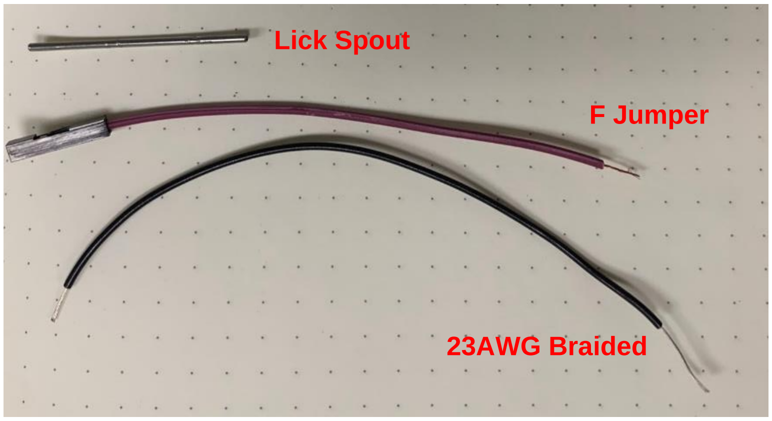

    c. Solder the stripped end of the F jumper wire to the 1cm of exposed wire on the 23AWG braided wire and check for a stable connection by firmly pulling from either end.

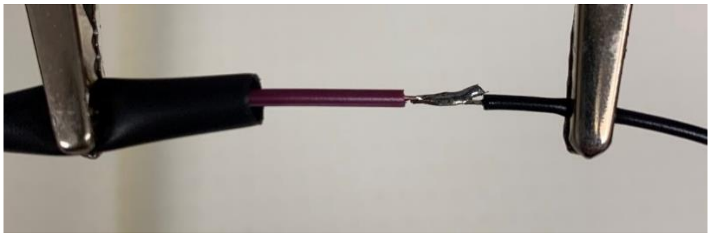

    d. Cover the exposed piece of newly joined wire with heat shrink tubing and heat with a heat gun until snug.

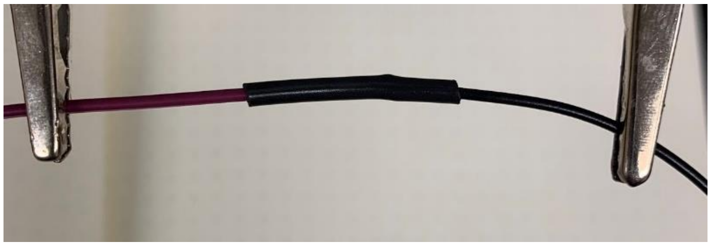

2) Solder each ***Spout*** to a wire
    a. Put on protective eyewear.
    b. Mount the Dremel with cutting disc to vice clamp and turn on.
    c. Score the circumference of the ***Spout*** ~1.5cm from one end of the ***Spout*** by gently pressing the ***Spout*** to the cutting disc and rotating the ***Spout*** with your hands.
    d. Separate and wrap the exposed 2cm of 23AWG wire around the scored portion of the ***Spout*** as tightly as possible. Ideally, the individual strands should be interwoven.

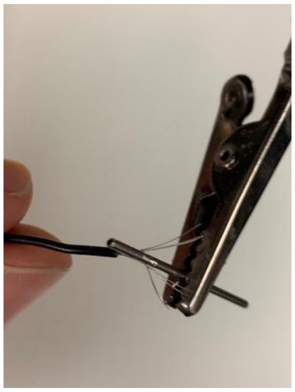
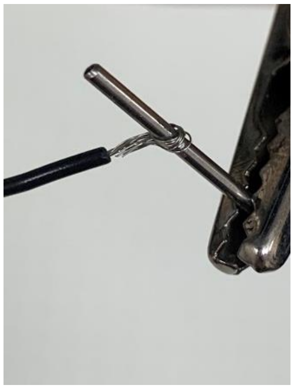

e. Mount the ***Spout***/wire to the helping hands.
f. Use the soldering iron to heat up the ***Spout*** /wire for an extended period and then encase the ***Spout***/wire junction with solder.

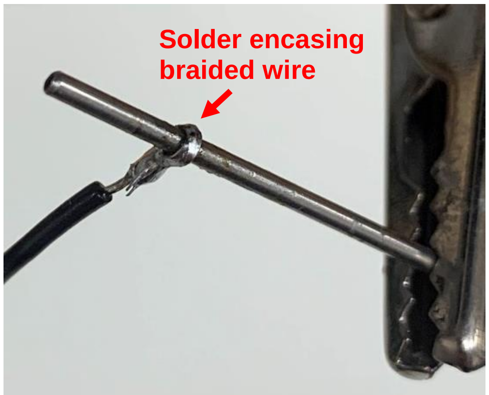

g. Allow the solder to cool completely and then ***check the strength of the connection by attempting to rotate the wire forcefully***. If the wire can rotate or slide, repeat the previous step until the connection is secure.
h. Once you have confirmed the wire to ***Spout*** connection is solid, strengthen the junction by placing a small segment of heat shrink tubing over the junction and heat with a heat gun until snug.

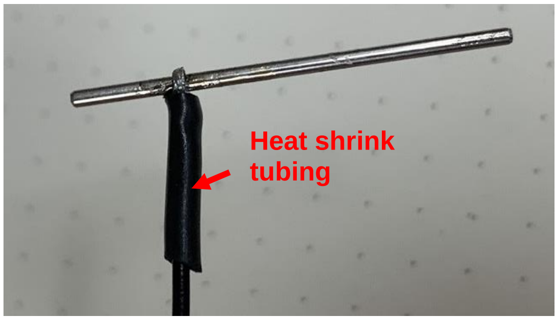

3) Press one end of the angled barb connector onto the ***Spout*** on the end closest to the wire until the ***Spout*** is inserted past the barb of the connector. Rotate so the open barb is in parallel with the wire.

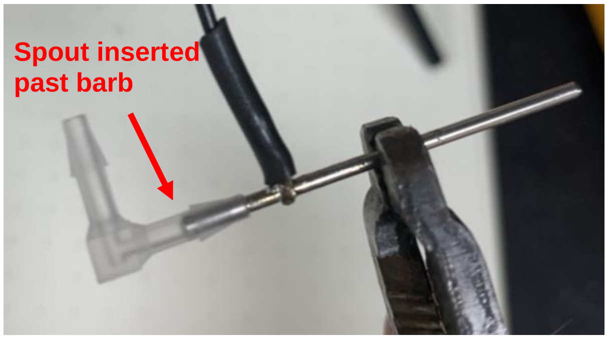

4) Fix ***Spout Assemblies*** in position.
   a. Set the ***Multi-Spout Head*** in the center of the ***Multi-Spout Head Assembly Guide***. And rotate so the guide holes are facing twoards the arc with the spout inset.
   b. Insert each ***Spout*** through each guide hole in the ***Multi-Spout Head*** and then continue to insert until the ***Spout*** slots into the spout inset of the ***Multi-Spout Head Assembly Guide*** and gently touches the wall at the back of the inset. If the fit is too tight, use an 18-gauge needle to expand the hole by pushing the needle through while rotating.
   c. Rotate the ***Spout Assemblies*** so the wire and barb are pointed upward. And check to ensure that vertical barbs are not touching.

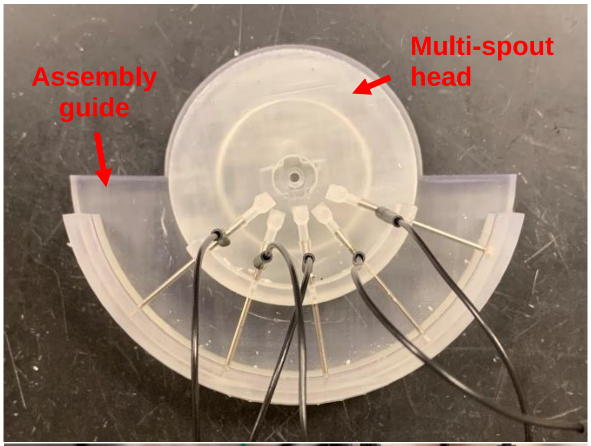

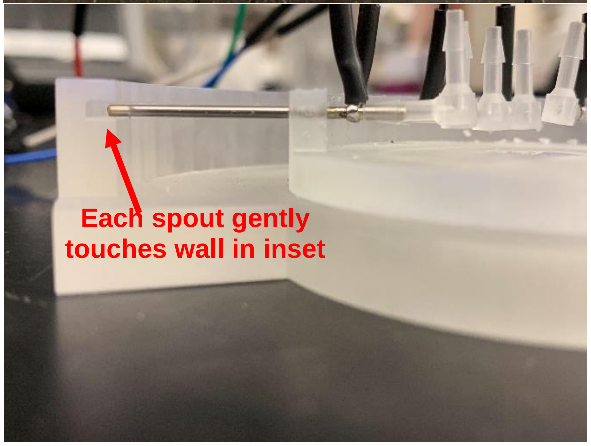

   d. Apply super glue to the junction between each ***Spout*** and the guide hole of the ***Multi-Spout Head*** on the inside and outside.

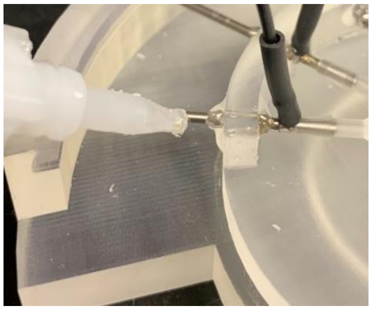

e. Allow the assembly to cure until super glue is completely set.
f. Rotate ***Multi-Spout Head*** to remove from ***Multi-Spout Head Assembly Guide***.

*Note: If you do not use the **Multi-Spout Head Assembly Guide**, make sure that each **Spout** extends out of the **Multi-Spout Head** to the same length and that each **Spout** is tilted to the same height. Differences in length can be corrected using the **Linear Actuator**, differences in height cannot be corrected using our hardware design.*

Linear Actuator (single spout)

Components and building materials:

- 3D printed components
  - ***Rack Body***
  - ***Rack Spur***
  - ***Helical Gear***
  - ***P-Body***
  - ***Cover***
- Electrical components
  - ***Servo***
- Assembled component
  - ***Spout***
- Tools and Materials
  - Philips screwdriver
  - Razor blade
  - Machine screws (4x)
  - Hex nuts (4x)
  - Screws for ***Servo*** (2x large, 1x small, should be included with ***Servo***)
  - 4 arm horn for ***Servo*** (should be included with ***Servo***)
  - Machine oil
  - Vice clamp
  - Dremel with cutting disc
  - Straight barb connector

<u>Instructions:</u>

1) Secure the ***Servo*** to the ***P-Body*** using the 2 large screws that came with the ***Servo***. Make sure that the ***Servo*** is oriented with the output shaft of the ***Servo*** centered in the circular cut out of the ***P-Body***.

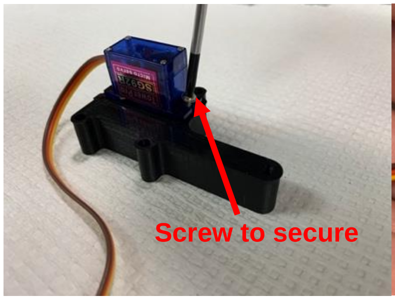

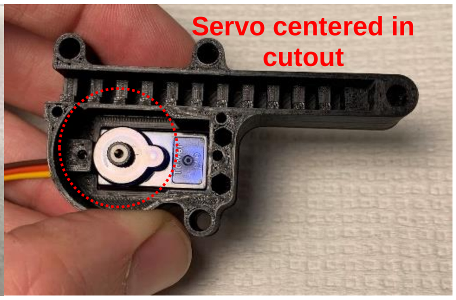

2) Score the horn by pressing and wiggling the sharp edge of a razor at the second hole from the center at an angle perpendicular to the arm. Next, hold the horn and forcefully bend the arm to snap the horn at the scored position.

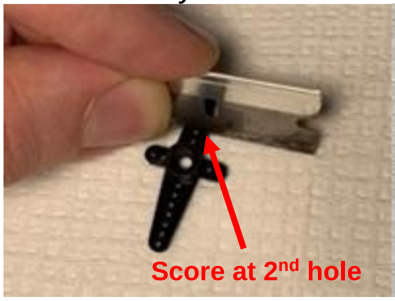

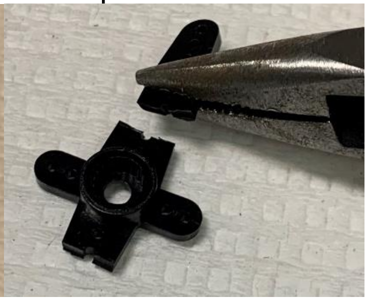

3) Seat the horn in the ***Helical Gear*** with the cylindrical extension of the horn facing away from the opening of the ***Helical Gear***. If the horn does not fit, repeat the previous step to make the horn shorter.

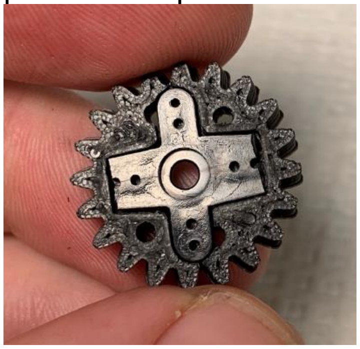

4) Attach the horn/***Helical Gear*** to the output shaft of the ***Servo*** by forcefully pinching the horn to the ***Servo*** with your fingers and then screwing into place using the small screw that came with the ***Servo***.

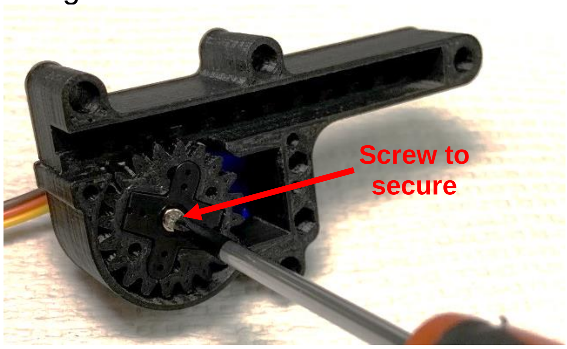

5) Insert the ***Rack Spur*** into the ***Rack Body*** with the spout mount and the spurs facing in opposite directions.

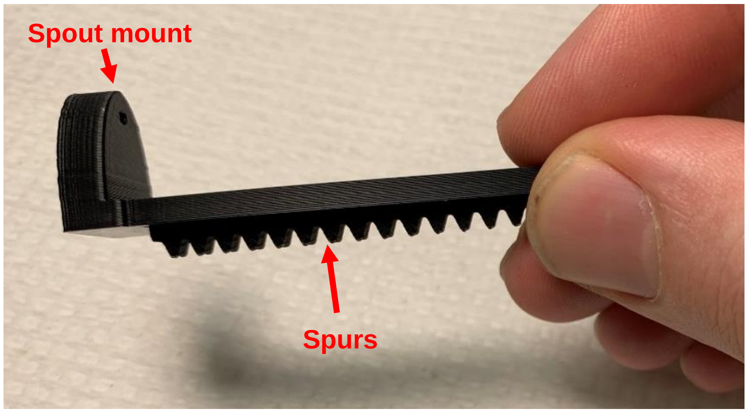

6) Use machine oil to liberally lubricate the ***Rack Spur***, ***Helical Gear***, ***P-Body***, along the inset, and ***Cover*** along the inset.
7) Rotate the gear **CLOCKWISE** as far as possible using the Rack Spur and remove ***Rack Spur***.
   *Note: If **Servo** will not rotate with modest force, connect the **Servo** to an Arduino and manually set the position of the **Servo** to 0.*
8) Insert the ***Rack Spur*** / ***Rack Body*** into the ***P-Body*** with the rear ***Rack Body*** as close to the back of the ***P-Body*** as possible. The teeth of the ***Rack Spur*** should thread between the teeth of the ***Helical Gear***.

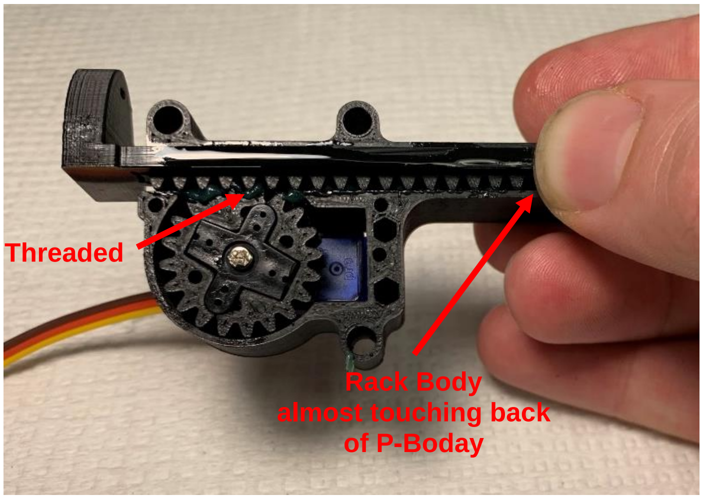

9) Place the ***Cover*** onto the ***P-Body***.

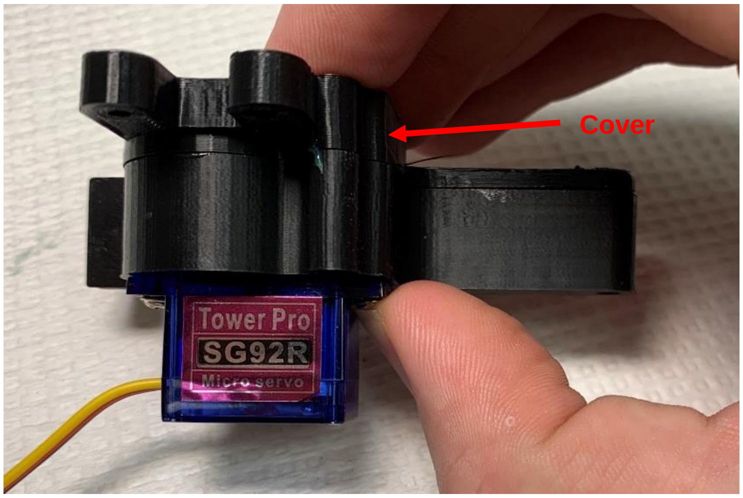

10) Cut machine screws to be long enough to fit through the ***P-Body*** / ***Cover*** and attach a hex nut.

   a. Mark the intended length on the machine screw using a felt tipped pen.
   b. Mount the machine screw in the vice clamp on the terminal end of the screw leaving the length marker exposed.
   c. Use the Dremel to cut the machine screw. The screw will become very hot, so use forceps or another tool to handle the screw after cutting.
   d. Test that the threads of the machine screws have not been damaged by ensuring a hex nut can still screw onto the end.

Measuring length for screw

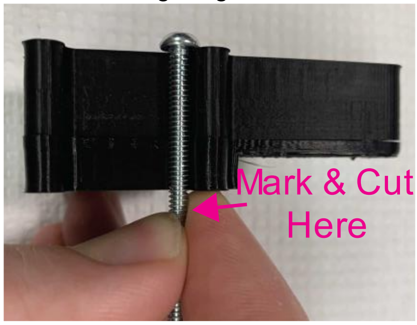

Cutting screw in vice clamp

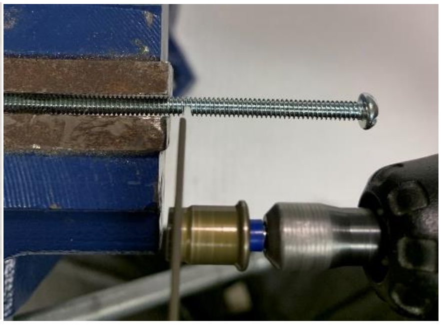

11) Insert machine screws through the 4 screw holes in the ***P-Body*** / ***Cover*** and screw hex nuts onto the extended portion of the machine screws.

Front

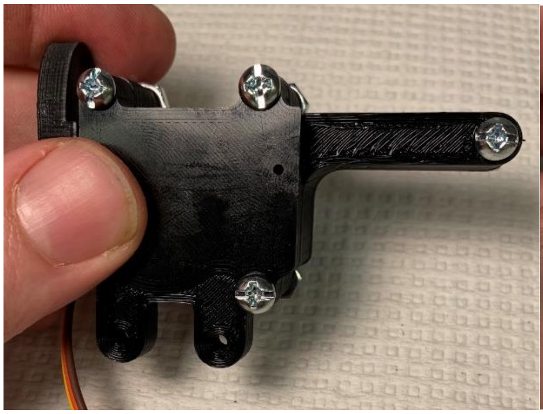

Back

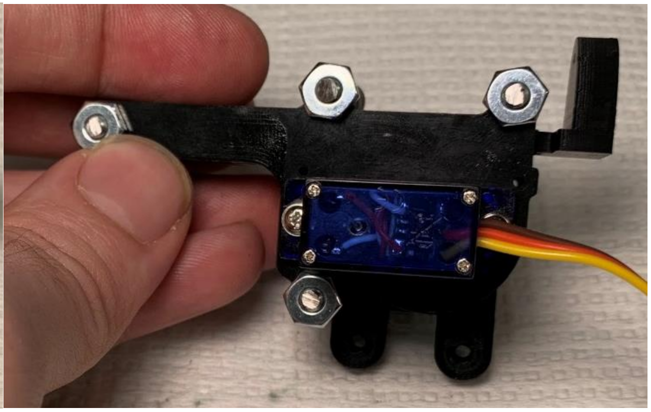

Linear Actuator & radial actuator (multi-spout)

Components and building materials:

- 3D printed components
  - ***Rack Body***
  - ***Rack Spur***
  - ***Helical Gear***
  - ***P-Body***
  - ***Multi-Spout Cover***
- Electrical components
  - ***Servo*** (x2)
- Assembled components
  - ***Multi-Spout Head*** with ***Spouts*** attached (see above)
- Tools and Materials
  - Philips screwdriver
  - Razor blade
  - Machine screws (4x)
  - Hex nuts (4x)
  - Screws for ***Servo*** (4x large, 2x small, should be included with servo)
  - 4 arm horn for servo (2x, should be included with servo)
  - Machine oil
  - Vice clamp
  - Dremel with cutting disc

*See photos from **Linear Actuator** (single spout) for reference*

Instructions:

1) Secure the first ***Servo*** to the ***P-Body*** using the 2 large screws that came with the ***Servo***. Make sure that the ***Servo*** is oriented with the output shaft of the ***Servo*** centered in the circular cut out of the ***P-Body***.
2) Secure the second ***Servo*** to the ***Rack Body*** using the 2 large screws that came with the ***Servo***. Make sure that the ***Servo*** is oriented with the output shaft of the ***Servo*** centered with the parallel arms of the rack mount.
3) Score the horn by pressing the sharp edge of a razor at the second circular hole into the arm perpendicular to the arm. Next, hold the horn and forcefully bend the arm to snap the horn at the scored position. Repeat for the second horn.
4) Seat one of the horns in the ***Helical Gear*** with the extension of the horn facing away from the opening of the ***Helical Gear***. If the horn does not fit, repeat the previous step to make the horn shorter.
5) Attach the horn/***Helical Gear*** to the output shaft of the ***Servo*** by forcefully pinching the horn to the ***Servo*** with your fingers and then screwing into place using the small screw that came with the ***Servo***.
6) Insert the ***Rack Spur*** into the ***Rack Body*** with the spout mount and the spurs facing in opposite directions.
7) Liberally lubricate the ***Rack Spur***, ***Helical Gear***, ***P-Body***, and ***Cover*** with machine oil.
8) Rotate the gear **COUNTERCLOCKWISE** as far as possible.

9) Insert the ***Rack Spur***/***Rack Body*** into the ***P-Body*** with the front ***Rack Body*** as close to the inside edge of the ***Multi-Spout Head*** as possible. The teeth of the ***Rack Spur*** should thread between the teeth of the ***Helical Gear***.
10) Place the ***Cover*** onto the ***P-Body***.
11) Cut machine screws to be long enough to fit through the ***P-Body*** / ***Cover*** and still attach a hex nut (~3cm).
    a. Mark the intended length on the machine screw using a felt tipped pen.
    b. Mount the machine screw in the vice clamp on the terminal end of the screw leaving the length marker exposed.
    a. Use the Dremel to cut the machine screw. The screw will become very hot, so use forceps or another tool to handle.
    c. Test that the threads of the machine screws have not been damaged by ensuring a hex nut can still screw onto the end.
12) Insert machine screws through the screw holes in the ***P-Body*** / ***Cover*** and screw hex nuts onto the extended portion of the machine screws.
13) Rotate the ***Servo*** mounted in the ***Rack Body*** **CLOCKWISE** as far as possible.
14) Seat the second horn in the ***Multi-Spout Head*** assembly with the left ***Spout*** as close to slightly clockwise of the axis of travel. Next fix the horn to the ***Servo*** by forcefully pinching the horn to the ***Servo*** with your fingers and then screwing into place using the small screw that came with the ***Servo***.

Wheel Brake

Components and building materials:

- Electrical component
    - ***Stage-to-Wheel-Brake-Servo-Adapter***
    - ***Brake Bar***
- Tools and Materials
    - Servo
    - Screws for ***Servo*** (2x large, 1x small, should be included with servo)
    - 1 arm horn for ***Servo*** (should be included with ***Servo***)

<u>Instructions:</u>

1) Secure the ***Servo*** to the ***Stage-to-Wheel-Brake-Servo-Adapter*** using the 2 large screws that came with the ***Servo***. Make sure that the ***Servo*** is oriented with the output shaft of the ***Servo*** located on the bottom of the adapter (single arm of the adapter is located on the bottom).

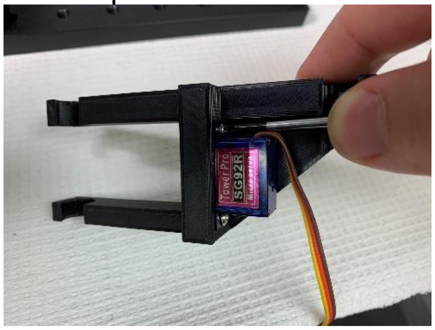
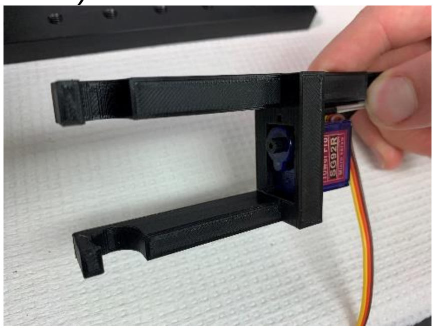

2) Attach the one-arm horn and rotate the ***Servo*** **CLOCKWISE** as far as possible.

*Note: If **Servo** will not rotate with modest force, connect the **Servo** to an Arduino and manually set the position of the **Servo** to 0.*

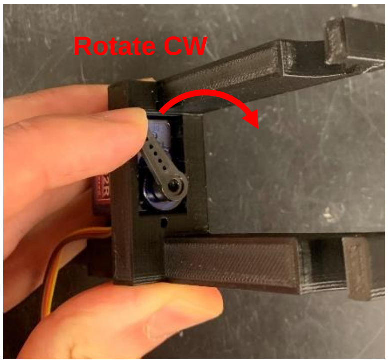

3) Remove the horn from the ***Servo*** and then re-attach at ~45-degree angle and then fix in place using the small screw included with the ***Servo***.

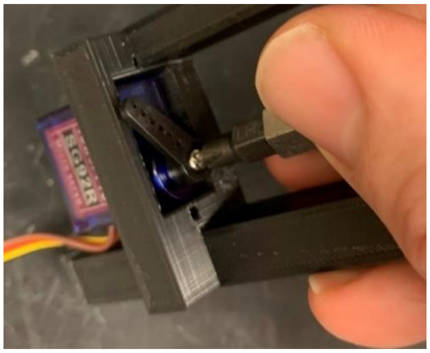

4) Place the ***Brake Bar*** onto the extension of the horn using moderate force. With the ***Brake Bar*** attached, there should be only a slight gap between the ***Brake Bar*** and the ***Stage-to-Brake-Bar-Adapter*** (black 3D printed piece in photo). If there is a large gap or if you cannot fit the brake on without rotating the ***Servo*** counterclockwise, then remove the horn and reseat at a different angle.

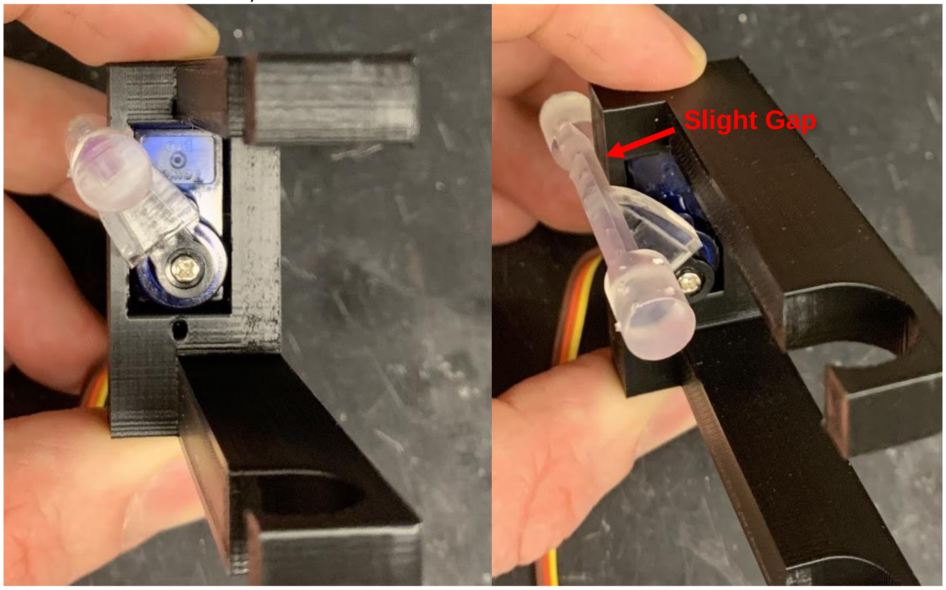

Head-Fixation Plate

Components and building materials:

- 3D printed components
    - ***Head-Fixation Plate***
- Tools and materials
    - Hex nut (2x)
    - Sandpaper or Dremel with sanding disk
    - Machine screw (2x)
    - JB weld epoxy
    - Cotton swab with wooden shaft

<u>Instructions:</u>

1) Rough up all sides and once face of each hex nut using sandpaper or Dremel.
2) Lay the ***Head-Fixation Plate*** upside down.
3) Use a wooden end of the cotton swab to thoroughly mix equal parts of the JB weld epoxy.
4) Apply a small amount of the epoxy to the hex inset on the underside of the ***Head-Fixation Plate***. Make sure to avoid getting epoxy in the screw hole located in the center of the hex inset.
5) Screw the machine screw into the smooth side of the hex nuts until it extends ~1cm. Carefully guide the screw into the hole and press the hex nut into the JB weld epoxy.
6) Repeat step 5 for the other screw/hex nut.
7) Use a wooden end of the cotton swab to carefully apply JB weld to the junction between the hex nut and the hex inset.

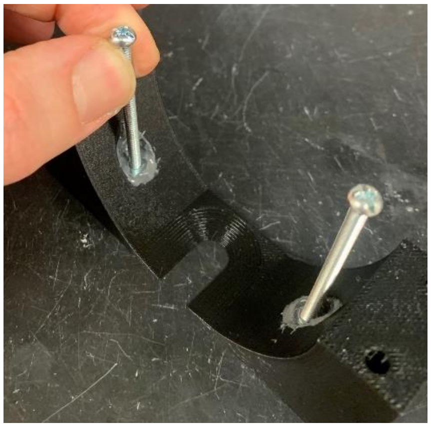

8) Leave the screws in place and allow the epoxy to cure overnight.
9) Remove the screw and check to ensure that the screw can easily rotate within the fixed hex nut. If you cannot remove the screw, you will need to remove the hex nut or get a new head-plate and start over.

Rotary Encoder

Components and building materials:

- Electrical component
    - Rotary encoder
- Tools and Materials
    - Helping hands
    - Heat gun
    - Soldering Iron
    - Solder
    - MM Jumper wires with unique colors including red and back (4x)
    - ~2cm small diameter heat shrink tubing (1/8”) (8x)
    - ~4cm large diameter heat shrink tubing (3/4”)

Rotary encoder connections:

- Ground (White) → Common ground
- 5V Power (Brown) → 5V power supply
- Rotary encoder A (Grey) → Arduino pin
- Rotary encoder B (Green) → Arduino pin

<u>Instructions</u>

Solder wires listed above to jumper wires.

1) Solder a male (M) pin of a custom jumper wire to the grey, green, white, and brown cables by applying solder to the M pin and the exposed wire and then joining the solder together. If possible, solder red to brown (5V), black to wuite (GND), grey to grey, and green to green (A).

2) Cover the exposed portion of the M pin/wire using small diameter heat shrink tubing (B) and heat with a heat gun until snug.

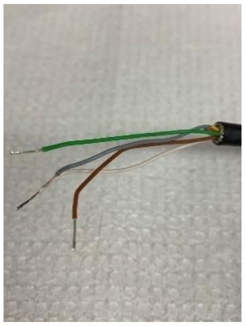

A

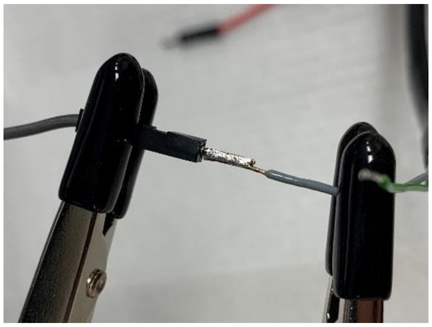

B

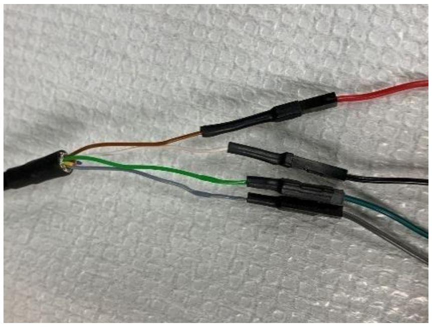

Isolate additional wires not listed above (red, yellow, blue, and pink)

3) Cut wires near the cable opening leaving ~1cm (A).
4) To prevent wires from contacting one another, cover exposed ends with small diameter heat shrink tubing and heat with a heat gun until snug.

5) To secure unnecessary wires, bend wires back and use large diameter heat shrink tubing to hold the wires against the black cable and heat with a heat gun until snug (B).

A

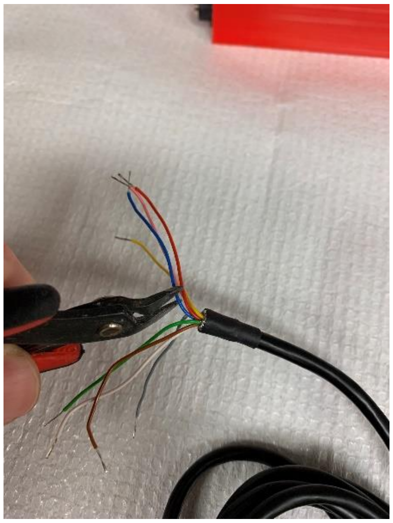

B

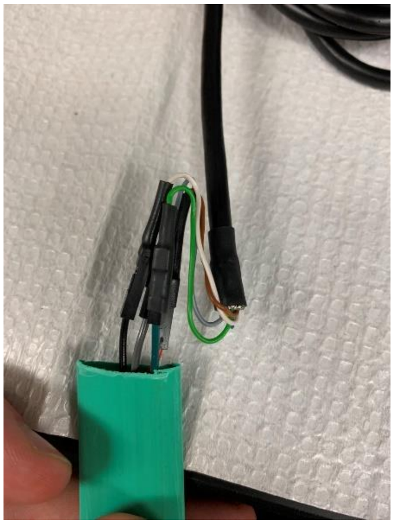

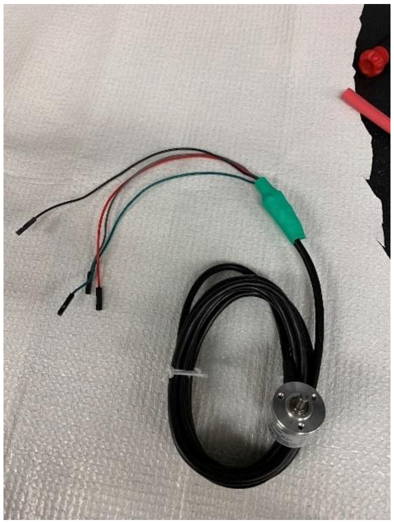

Capacitive Touch Sensor (MPR121)

Components and building materials:

- Electrical component
  - Capacitive touch sensor
- Tools and Materials
  - MM pin array (should be included with capacitive touch sensor)
  - Solder
  - Soldering iron
  - Solderless breadboard

<u>Instructions:</u>

1) Trim the 2 pin arrays to the number of leads on each end of the capacitive touch sensor (MPR121 2022 has 13 pins on one side and 7 pins on the other).
2) Set the capacitive touch sensor on the solderless breadboard to determine the location of bread board pin receivers that line up with the leads on the sensor.
3) Insert the long end of the pin arrays into the solderless breadboard receivers.
4) Seat the capacitive touch sensor on the short end of the pin arrays. The sensor should rest flush with the top of the plastic component of the pins and the pins should be straight.

Sensor seated on pins

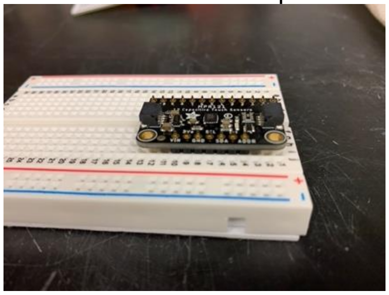

5) Apply solder to each of the lead and pin junctions
   a. Place the soldering iron at the junction between the lead and pin.
   b. Apply a small mound of solder to encase the pin making sure that adjacent pins/solder do not make contact. If you accidentally solder multiple pins together, use soldering wick to remove
   c. Repeat for all pins.

Soldering pins to sensor

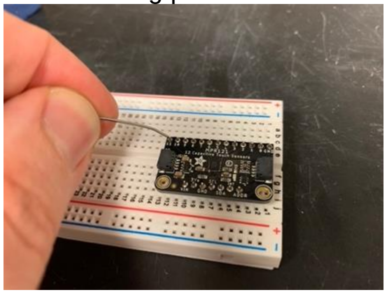

6) Pull the capacitive touch sensor with pins attached off the solderless breadboard.

Lick detector clip (single spout)

Components and building materials:

- Tools and Materials
  - Jumper wire (MF)
  - Alligator clip
  - Wire stripper
  - Soldering iron
  - Solder
  - Heat shrink tubing
  - Heat gun

Instructions:

1) Cut the alligator cable ~10cm from one of the alligator clips and strip the long wire leaving ~1cm of wire exposed. Set aside the other side of the alligator clip / wire.
2) Solder the M pin of the MF jumper wire to the ~1cm of exposed wire attached to the alligator clip.
3) Cover the exposed wire with heat shrink tubing and heat with a heat gun until snug.

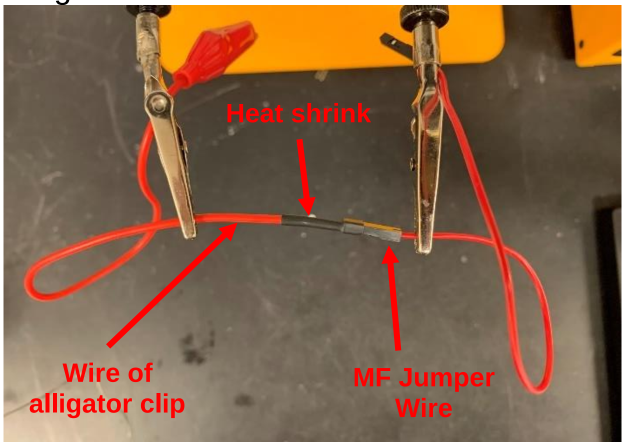

Solenoids

Components and building materials:

- Electrical component
  - ***Solenoid(s)***
- Tools and Materials
  - Helping hands
  - Heat gun
  - Soldering iron
  - Solder
  - Jumper wire (MM) for each ***Solenoid*** (2x / ***Solenoid***, preferably 1 color per ***Solenoid***)
    - *Optional: 2x custom jumper wire housing (Arduino end).*
  - ~2cm small diameter heat shrink tubing (1/8”) (2x / ***Solenoid***)

Instructions:

1) Solder the male end of the jumper wires to the exposed wire of the ***Solenoid***. Preferably use a unique color for each ***Solenoid*** (2 red wires for ***Solenoid*** 1, 2 green for ***Solenoid*** 2, etc.).
2) Cover the exposed portion of the pin/wire using small diameter heat shrink tubing and heat with a heat gun until snug.

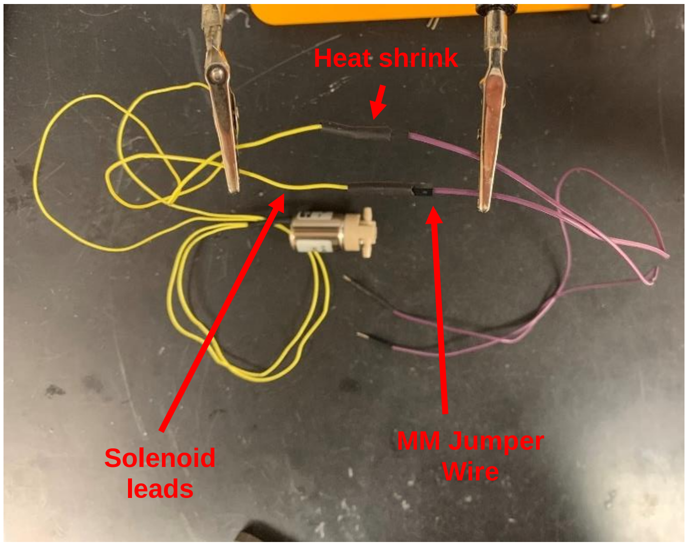

Tone Generator

Components and building materials:

- Electrical component
  - ***Tone Generator***
- Tools and Materials
  - Helping hands
  - Heat gun
  - Soldering iron
  - Solder
  - Jumper wires MM (2x, preferably red and black)
    - *Optional: 1x2 custom jumper wire housing (Arduino end).*
  - ~2cm small diameter heat shrink tubing (1/8”) (2x)

Instructions:

3) Solder the male end of the jumper wires to the exposed wire, keeping color consistent (Black to black, red to red).
4) Cover the exposed portion of the M pin/wire using small diameter heat shrink tubing and heat with a heat gun until snug.

Soldered leads prior to covering with heat shrink tubing

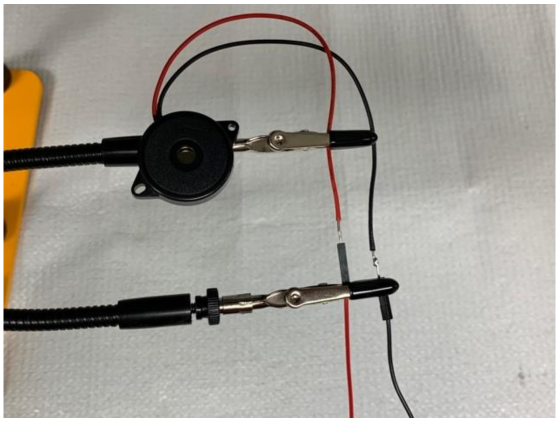

Assembly of Arduino Console

Components and building materials:

- 3D printed components
    - ***Arduino Case***
- Electrical component
    - ***Arduino Mega***
- Tools and Materials
    - Solderless bread board
    - Wire (23AWG solid)
    - Custom jumper wires (MF)
    - Custom jumper wires (MM)
    - Custom jumper wire housing
        - 1x4
        - 1x3
        - 2x8
        - 2x7
        - 2x2
        - 1x1
        - 2x6
    - Tip 120 (1/***Spout***)
    - 5.6kOhm resistor (1/***Spout***)
    - DC power pigtail (2x)
    - BNC female connector (4x, *optional*)
    - Nylon posts + hex nuts + screws (x2)
    - Wire stripper
    - Soldering iron
    - Solder
    - Heat shrink tubing
    - Heat gun
    - Labels (label maker, tape + sharpie, etc.)

Note: we are including instructions for the version of the ***Arduino Case*** *that includes custom jumper wire housing. The same system can be made using standard jumper wires or soldered wires. In our experience, the custom jumper wire housing approach is best at balancing speed with consistency, especially with novice users. It is absolutely crucial that the custom jumper housings are assembled with the proper organization of wires.*

Connectivity overview (reproduced from System Overview)

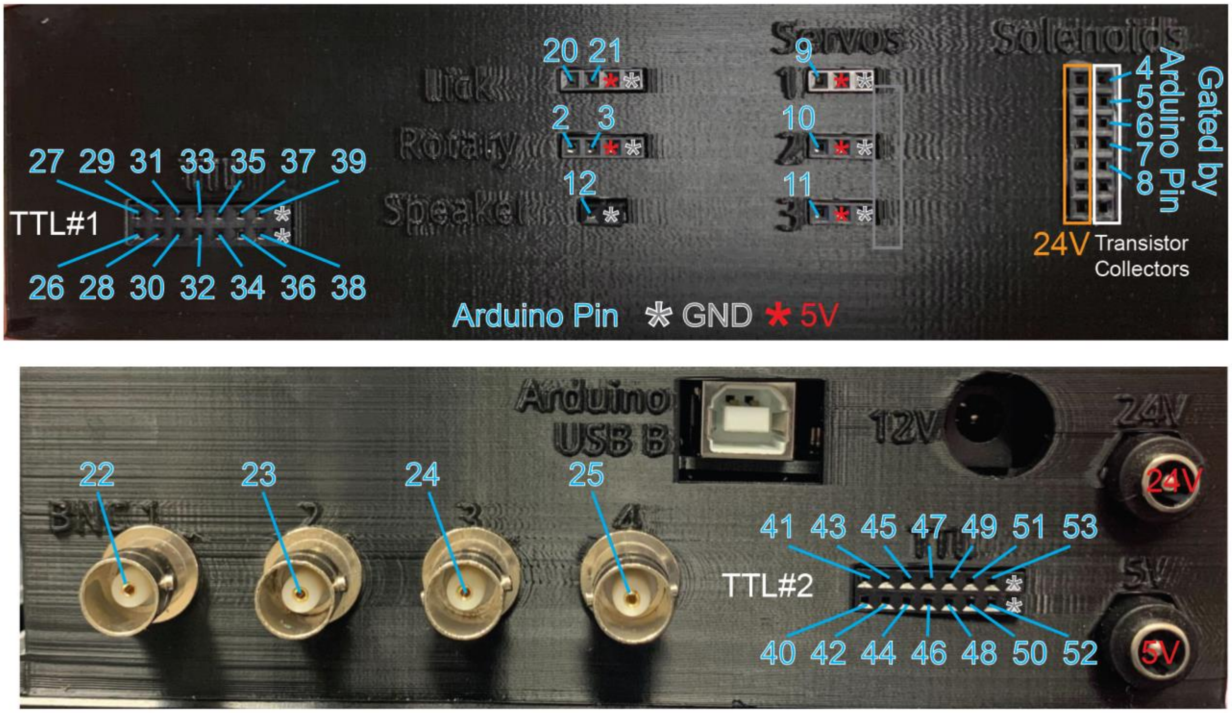

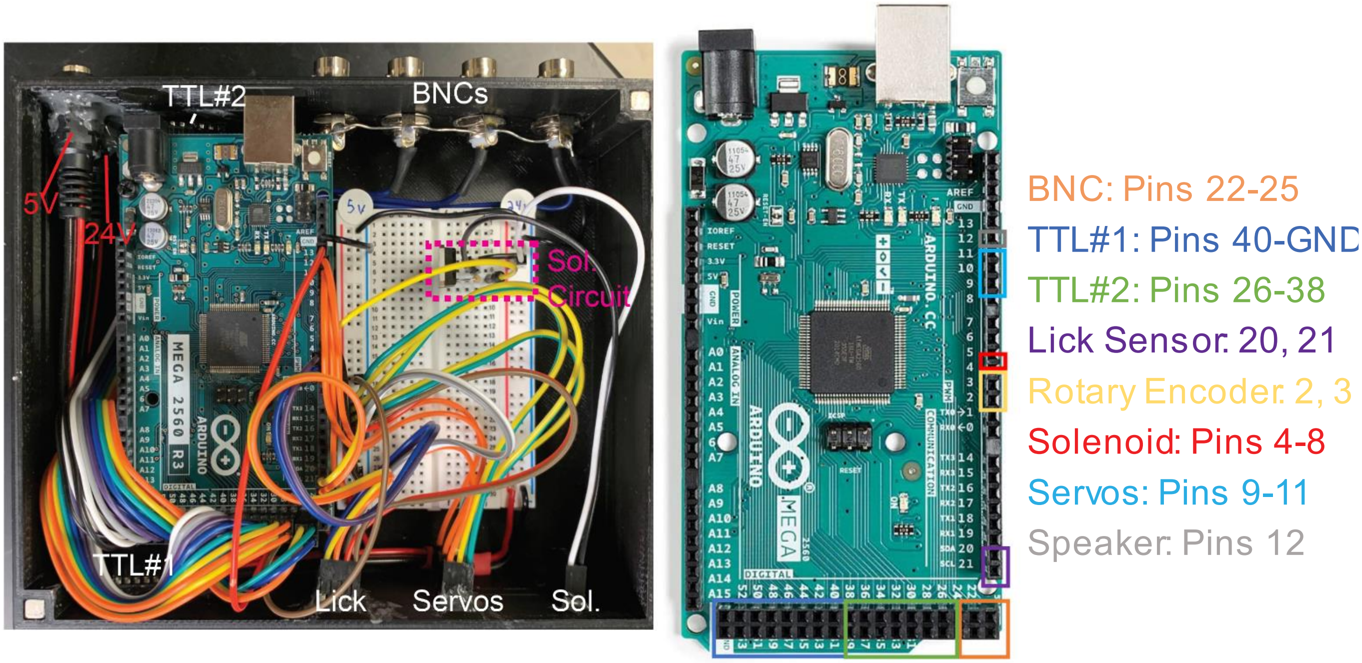

Refer to this image for connectivity of system

Instructions:

Arduino case

1) Secure the solderless breadboard by removing the film on the bottom of the board to uncover the adhesive surface. Press breadboard onto the flat surface adjacent to the ***Arduino*** (make sure to leave enough space to the right for the pigtail wires to fit and enough space to the left for the ***Arduino***).

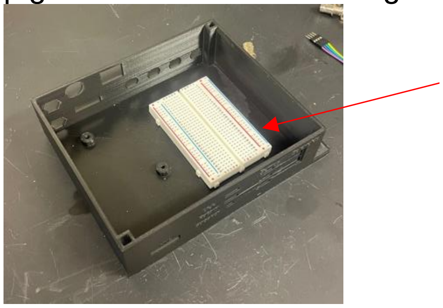

BNC connectors (optional)

2) Insert 4 female BNCs into the corresponding holes in the ***Arduino Case*** with leads facing inward.
3) Place a metal ring with grounding lead over the BNC connector from the inside of the box and use your fingers to gently tighten a hex nut on each BNC connector.
4) Use a pair of plyers to tighten the hex nuts to secure the female BNCs
    a. The metal grounding ring / leads tend to rotate a when the nuts are tightened so make sure the grounding lead remains facing towards the top.

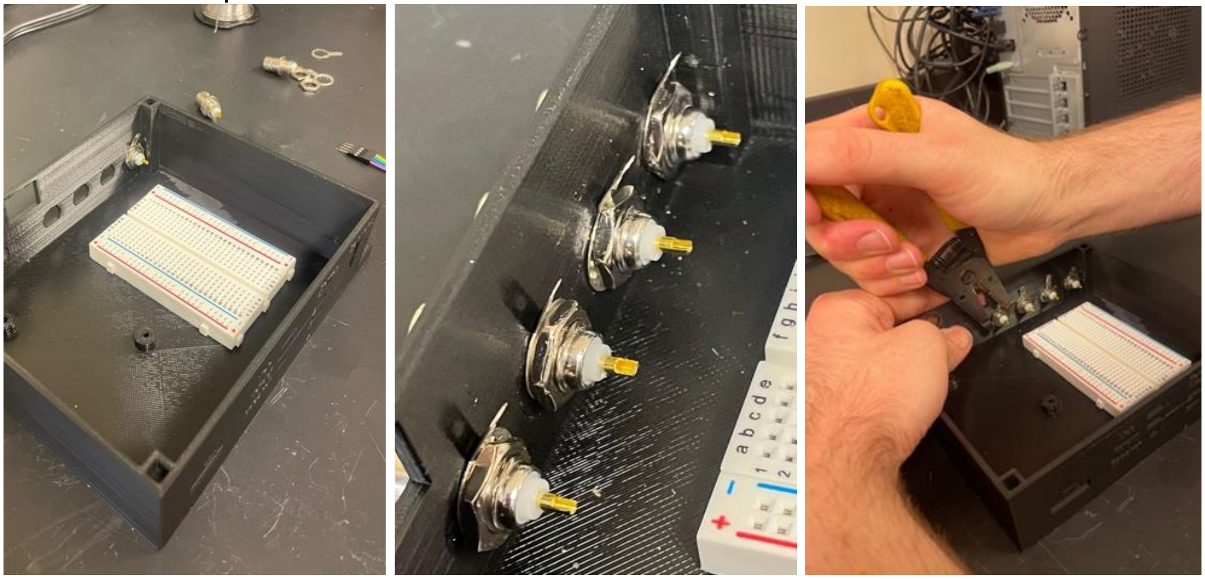

5) Prepare a MF- 1x4 / 1x4 custom jumper wire and lay in the ***Arduino Case***

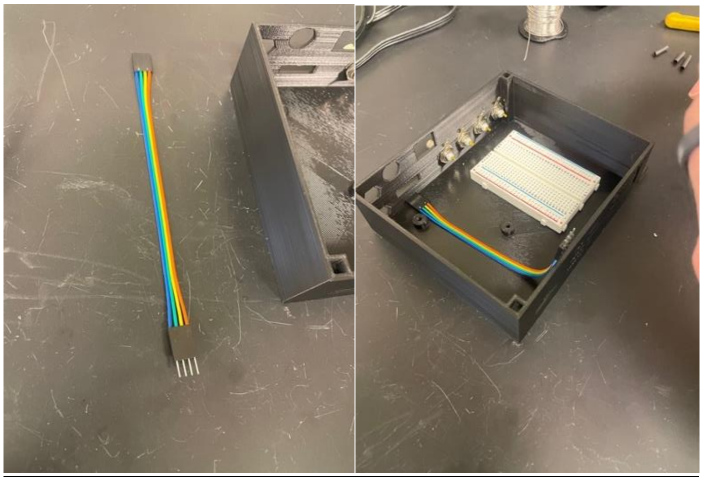

6) Using 23AWG wire, cut 4 pieces of a range of lengths with the longest being approximately 12.5-13cm and the shortest being approximately 5-6cm. Then strip both ends of the wires using wire strippers leaving ~1cm exposed.

Approximate length for the 4 wires

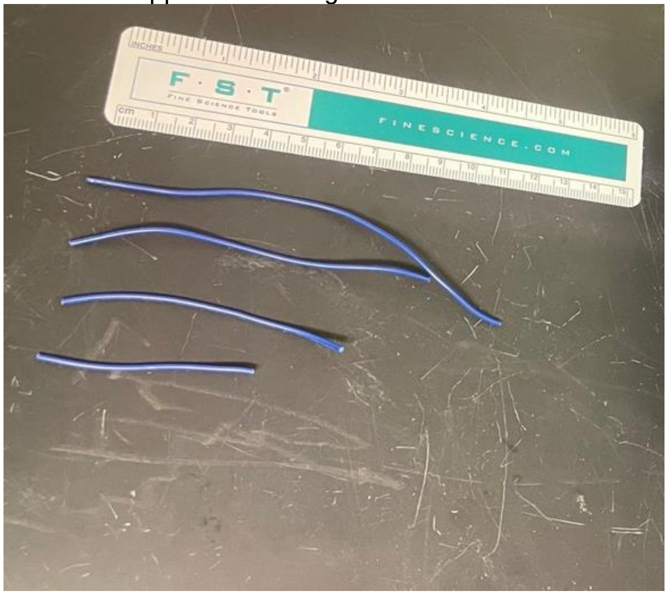

7) Add solder to each of the 4 BNC positive pins
    a. Heat up the cup using the soldering iron for several seconds, add solder into the cup making sure to melt the solder into the cup and not on the tip of the iron.

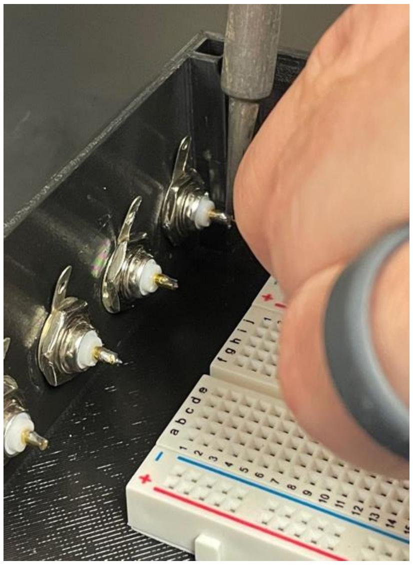

8) Solder the 23AWG wire to the male BNC pins
    a. Starting from the BNC on the right side, use the longest wire.
    b. Rest the wire on the solder within the BNC lead, then press a soldering iron against the wire until the wire heats up enough to melt and then sink into the solder.
    c. *Sanity Check:* If you added enough solder, you should be able to press your iron onto the stripped metal and it should slide into the solder in the golden cup in less than 10 seconds. If the solder in the cup is not melting in a few seconds, add more solder and repeat.
    d. Repeat steps above for the remaining BNCs, decreasing in wire length as you go from right to left.

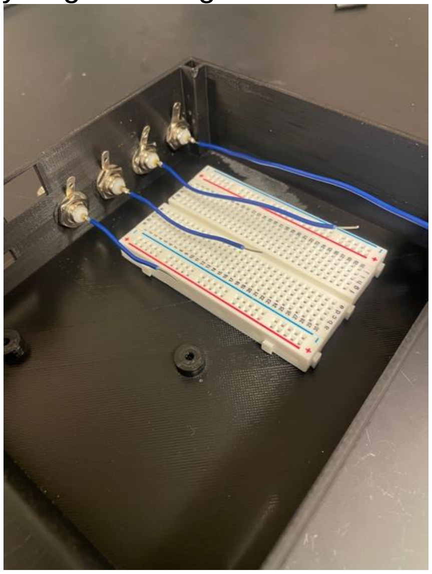

9) Use heat shrink tubing to cover the exposed region of the BNC / wire and use a heat gun or soldering iron heat until tubing is snug. *Warning: do not heat up the **Arduino Case** to the point it begins to melt; be cautious with how long the heat gun is pointed into the case.*

10) Connect BNC connectors together for grounding.

   a. Cut a length of black wire ensuring it is long enough to contact each BNC grounding lead and connect into the breadboard. Strip one end with ~1cm exposed and the other end with ~10cm (long enough to contact each BNC grounding lead).

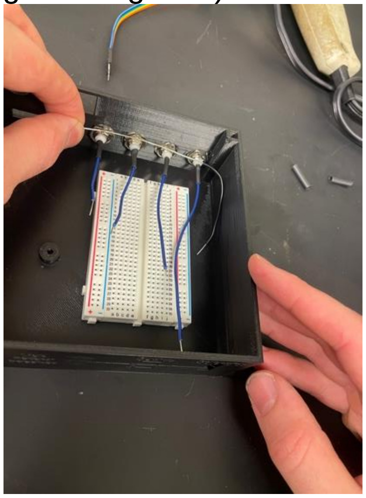

   a. Starting from the rightmost BNC, thread the end of the wire with the longer length exposed through the hole of each of the BNC grounding leads, and then wrap the end of the wire back around the last lead.
   b. Solder each junction between the wire and the BNC grounding leads with enough solder to encase the wire. Check the strength of each soldered point by firmly pulling on the wire.
      Note: if your BNC grounding leads do not have holes, solder each connection one at a time for each BNC from left to right.

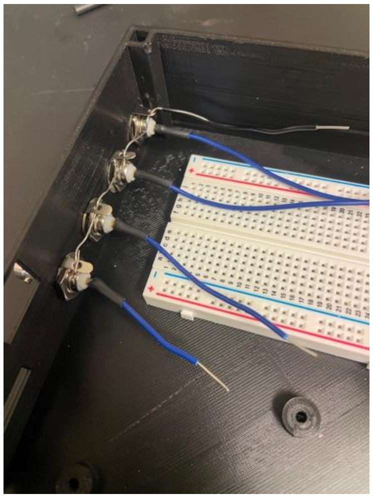

   c. Plug the opposite end of the grounding wire (the side with ~1cm exposed) into the right negative column on the solderless breadboard.
   d. Next, plug in each of the 23AWG wires into the F ports of the 1x4 custom jumper wire, making sure to maintain the order of the BNC connectors.

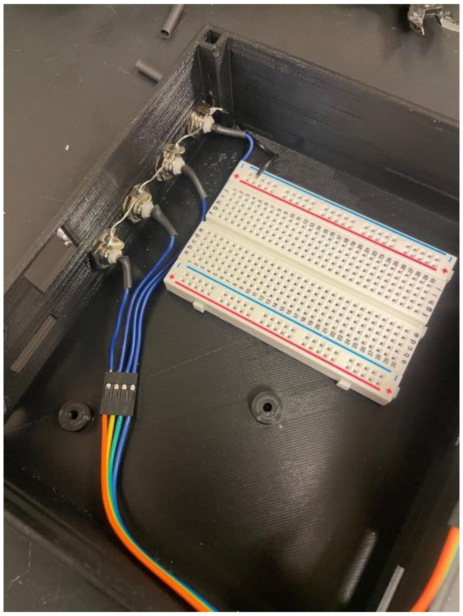

Power

1) Label the left pair of columns of the solderless breadboard with “5V” and the right pair of columns with “24V”.

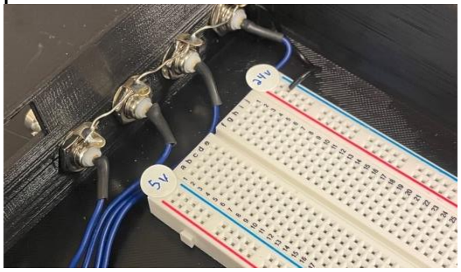

2) Take the 2 DC power pigtails and strip the red and black wires so ~1cm of wire is exposed. Firmly twist each exposed wire individually to reduce fraying of the individual strands.
3) Cut 2 lengths of ~3cm of black 23AWG wire, and 2 lengths of ~4cm of red 23AWG wire, then strip both ends leaving ~1cm of exposed wire.
4) Add solder to each of the pigtail wires (2 black, 2 red). Use solder to connect each pigtail wire to a corresponding length of 23AWG wire, making sure to keep color coding consistent (red to red, black to black).
5) Slide on a length heat shrink tubing long enough to cover the length of wire exposed at each connection point between the 23AWG wire and the pigtail wire and heat with a heat gun until snug. Add on a large diameter length of heat shrink tubing to cover the connection point wires of each pigtail and heat with a heat gun until snug

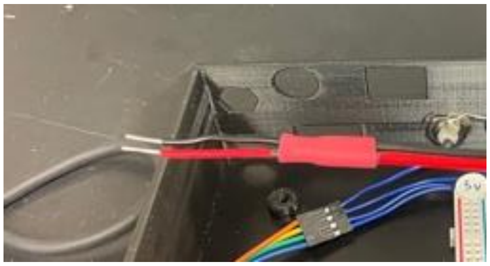

6) Slide the connector end of each of the DC power pigtails into the hexagonal holes on the upper left side of the box (labeled 24V (top) and 5V (bottom) on the face of the ***Arduino Case***).

Face of ***Arduino Case***

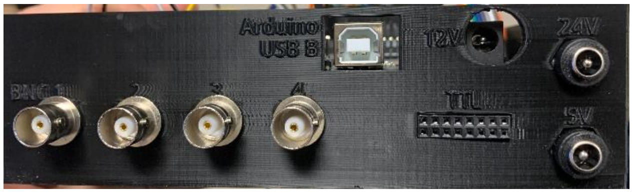

7) Next, plug the 1cm of exposed wire from each pair of pigtails into the corresponding power columns on the solderless breadboard (black wire to the (–) column, red wire to the (+) column). Make sure that the DC power pigtail mounted in the 5V position is connected to the 5V columns, and that the one mounted in the 24V position is connected to the 24V columns.
8) Ground the two (-) columns on the solderless breadboard together by cutting a ~6cm length of wire, stripping ~1cm on both sides, and then plugging the wire into both (-) columns. (*Note: you can also use a MM jumper wire for this*)

Final Product

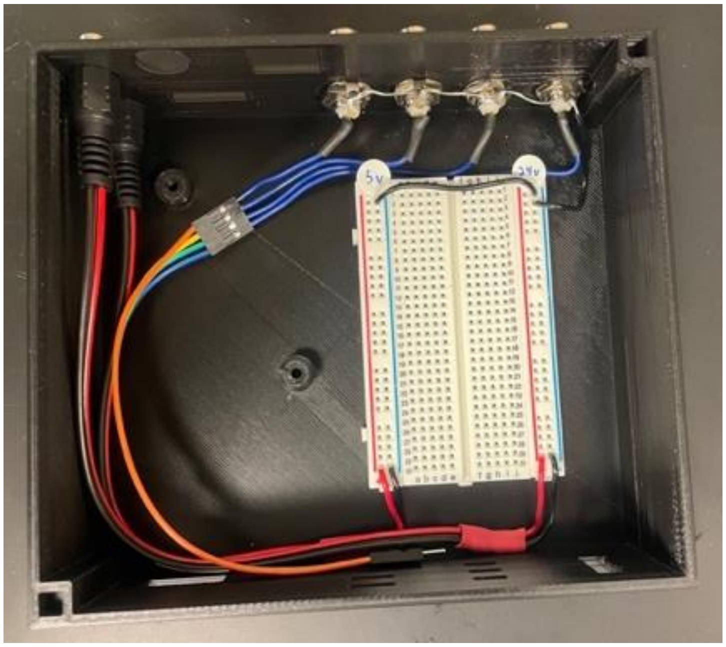

General organization of external connectors:

Rightmost connector = GROUND
Second to right connector = (+)

External connectors: TTL

- **TTL2** (port on back of ***Arduino Case*** with BNC connectors)
    a. Grab 2 sets of 8 custom M/F jumper wires (black to orange (x2) so you should have 16 wires).
    b. Plug female pins of those 16 cables into a 2x8 custom housing. Plug male pins into a second 2x8 custom housing.
        i. Maintain the order of the ribbon of jumper wires (Black → orange) on both rows of the custom jumper wire housing. The top and bottom row the housing should have colors mirrored.

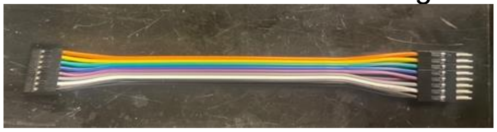

    c. Press jumper housing with F connectors into the hole within the housing labeled TTL with the black side of the ribbon located on the right side of the hole when looking at the outside face of the ***Arduino Case***. Leave jumper wires across the ***Arduino Case*** (see image below).

2) **TTL1** (port on front of ***Arduino Case***)
    a. Grab 2 sets of 8 custom M/F jumper wires (black to orange (x2) so you should have 16 wires).
    b. Plug female pins of those 16 cables into a 2x8 custom housing. Plug male pins from orange to white into a 2x7 custom housing, plug remaining 2 pins for into a 1x2 custom housing.
        i. Follow order for wires outlined in TTL1 section above

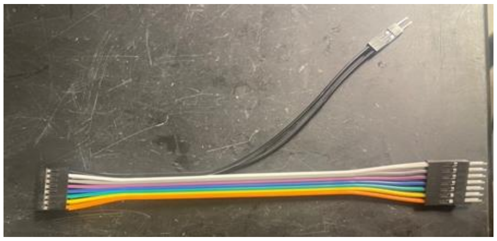

    c. Press jumper housing with F connectors into the hole within the housing labeled TTL with the black side of the ribbon located on the right side of the hole when looking at the outside face of the ***Arduino Case***. Leave jumper wires bent twoards the front of the ***Arduino Case*** (see image below).
    d. Plug the M pins of the black wires in the 1x2 custom housing into the (-) column of the 5V power columns

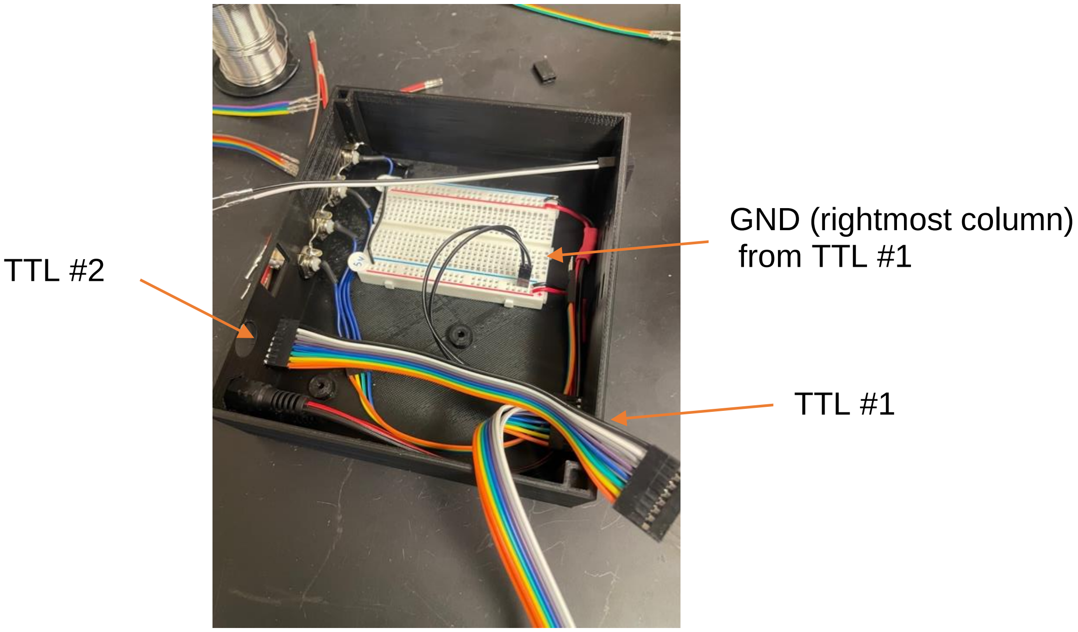
GND (rightmost column)
from TTL #1
TTL #2
TTL #1

Servos

1) Grab 3 sets of M/F custom jumper wires, preferably with the same colors in each set (orange – green in our example).
2) Plug the female pins of each set into a 1x3 custom housing while maintaining the order of the ribbon.
3) Insert the sets of 1x3 custom housing into the holes labeled "Servos 1-3" with the ground on the rightmost side (green in our example, *Note: the photos have the 1x3 custom housings in the opposite orientation*).
4) For the male pins, separate one of the side wires from the other two (orange in our example).
    a. Plug the single wires into a 1x3 custom housing maintaining the order of the holes in the case (top, middle, bottom).

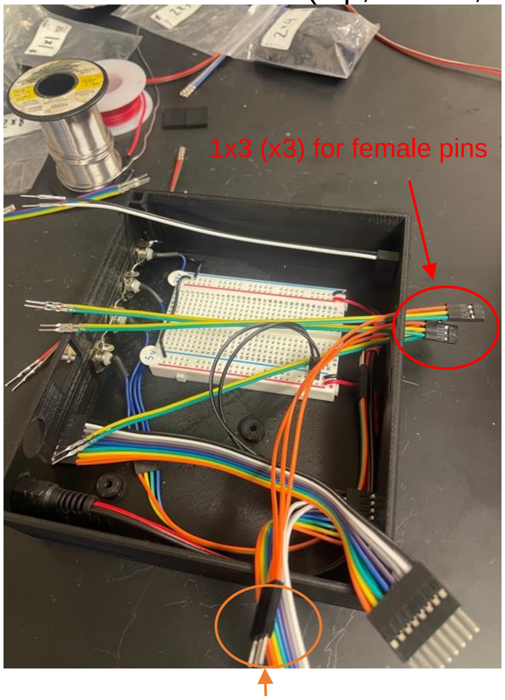

    b. Plug the dual wires into a 2x5 custom housing such that each color aligns with a single column (in our example: yellow in one column, green in the other).
    Note: this 2x5 housing will contain the ground (rightmost) and positive (second to right) M pins for all 3 ***Servos****, the capacitive touch sensor, and the* ***Tone Generator****.*

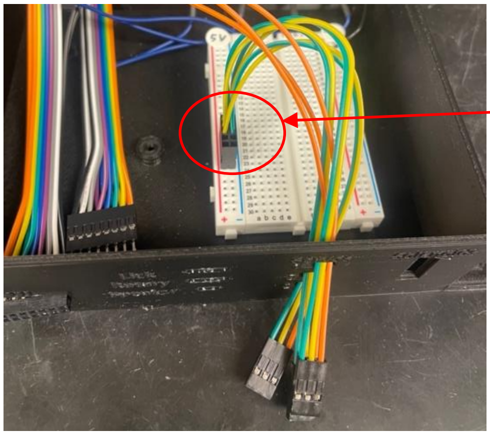

1x5 housing

- Middle M pin in left column (yellow)
- Right M pin in right column (green, 1x3 *housings are flipped in photo*)

Capacitive Touch Sensor (Lick)

1) Get 4 custom MF jumper wires (color does not matter).
2) Plug the female pins into a 1x4 pin-housing connector.
3) Insert the 1x4 custom housing into the hole labeled "Lick" with the ground on the rightmost side.
4) For the male pins, divide the 4 wires into 2 groups of 2.
    a. The right two 2 wires will go into the power 2x5 housing (should contain servo pins already plugged in). Plug the M pin for the wire from the right most position into the column of the 2x5 housing that contains grounding wires from the ***Servos*** (green in our example). Plug the M pin for the wire from the second to right position into the other column (yellow in our example).
    b. Plug the left two wires into a 1x2 custom housing.

Rotary encoder (Rotary)

1) Get 4 custom MF jumper wires (color does not matter).
2) Plug the female pins into a 1x4 pin-housing connector.
3) Insert the 1x4 custom housing into the hole labeled "Rotary" with the ground on the rightmost side.
4) For the male pins, divide the 4 wires into 2 groups of 2.
    a. The right two 2 wires will go into the power 2x5 housing (should contain servo pins already plugged in). Plug the M pin for the wire from the right most position into the column of the 2x5 housing that contains grounding wires from the ***Servos*** (green in our example). Plug the M pin for the wire from the second to right position into the other column (yellow in our example).
    b. Plug the left two wires into a 1x2 custom housing.
5) Plug the 2x5 housing that contains ground and positive pins for the ***Servos***, capacitive touch sensor, and the rotary encoder into the bread board power column labeled "5V" such that the rightmost pins for all the components is plugged into the (-) column

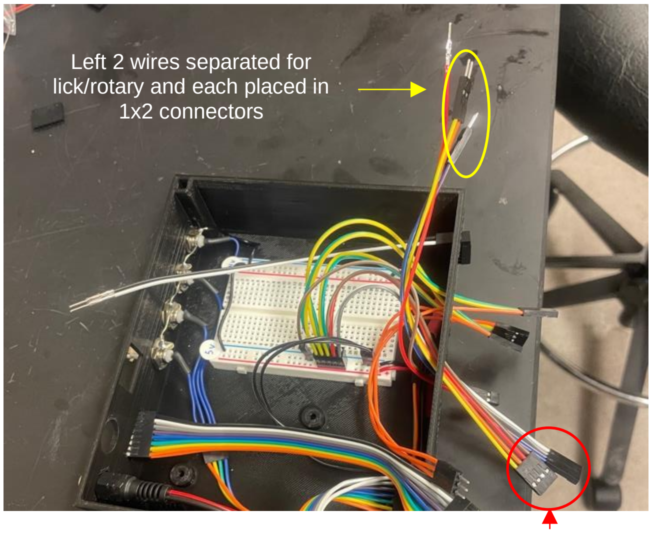

1x4 for rotary/lick

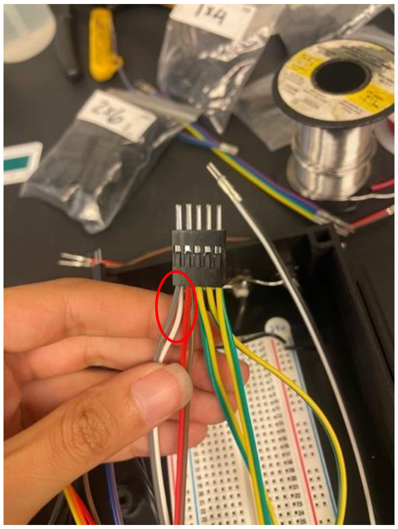

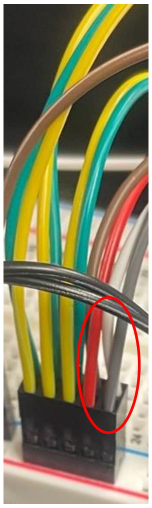

Right 2 wires in 2x5 connector

Solenoid(s)

1) Grab 2 custom M/F cables (color doesn't matter). Plug the female ends into the top row of a 1x7 custom housing and each of the male pins into 1x1 custom housings. Repeat for each ***Solenoid*** in the system, using a new row of the 1x7 custom housing.
2) Mount 1x7 in ***Arduino Case***.

Speaker

1) Grab 2 custom jumper wires (colors do not matter).
2) Plug the female pins into a 1x2 custom housing.
3) Plug each male pin into a separate 1x1 custom housing.
4) Insert the 1x2 custom housing into the hole labeled "Speaker".

Fix the Arduino into the box

1) Attach 2 nylon posts (2cm) to the base of the case by inserting the threaded side of the post through the bottom and rotating the post to screw on hex nuts placed on the bottom of the case. Depending on the 3D printer, you may need to manually remove some filament from the hex shaped insets (soldering iron can also work to melt filament).
2) Once the posts are secure, rest the ***Arduino*** on top of the posts and secure in place using nylon screws.

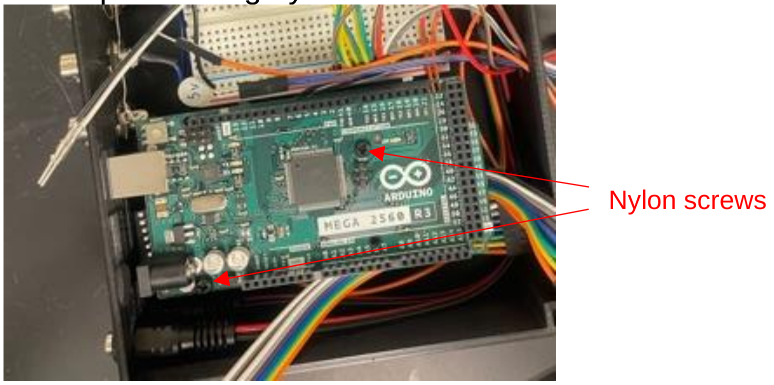

Connect wires to Arduino pins

Refer to key_arduinopins.xlsx for pin key

TTL #1: plug into pins 40-GND (black wires should be GND & GND).

TTL #2: plug into pins 26-38 (white wires should be 38 & 39).

Lick: plug into pins 20-21 (leftmost = 20, second to left = 21).

Rotary: plug into pins 2-3 (leftmost = 2, second to left = 3).

Speaker: left wire plugs into pin 12, right wire plugs into ground (-).

Servo: plug into pins 9-11 (Servo 1 = 9, Servo 2 = 10, Servo 3 = 11).

Ground: Use a MM jumper wire or strip of 23AWG wire to connect the ground of one of the rails with a ground on the Arduino.

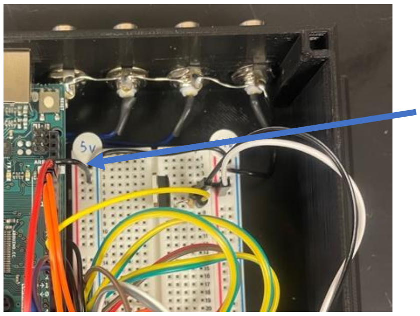

Black ground cable connected to ***Arduino***

Solenoid circuit

1) Attach the transistor in column **F** with the metal side of the transistor facing towards the middle of the bread board (which rows is unimportant).
2) Connect the common ground to the row with the ***right*** leg of the transistor (see photo below).
3) Connect a row of wires within the ***Solenoid*** 1x7 custom housing to
   i. 24V+ (red, left column in 24V power columns)
   ii. Column with the ***middle*** leg of the transistor
4) Connect the 24kOhm resistor in columns **G** and **I** in the row with the left leg of the transistor (see photo).
5) Connect an ***Arduino*** pin (4-8 for ***Solenoid*** 1-5, respectively) in column **I** in the row with the left leg of the transistor along with the resistor (see photo).
6) Repeat steps 1-5 for each ***Solenoid***, leaving at least 1 row between each ***Solenoid*** circuit.
   *Note: after completing **Solenoid** circuits check to ensure that the metal portion of the transistors are not touching each other.*

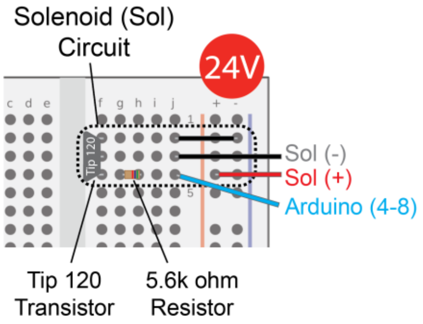

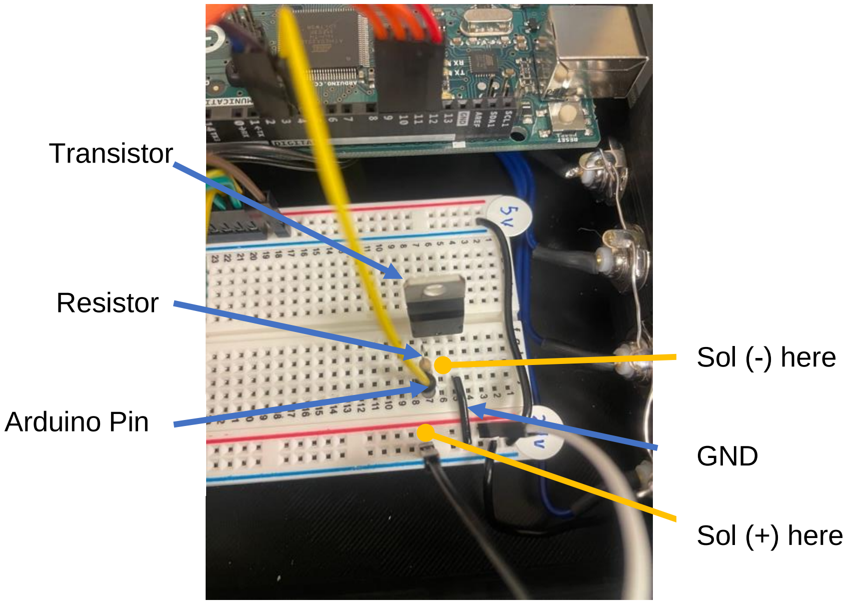

Epoxy ports

1) Mix equal volumes of the two components of JB weld with the wooden end of a cotton swab to create the epoxy (should look grey).
2) Make an application tool by snaping the wooden shaft of a cotton swab to create a tapered splinter.
3) Carefully spread epoxy over the junctions between each custom housing and the ***Arduino Case***.

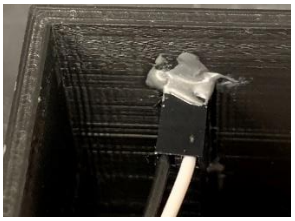

4) Do the same for the junctions between each DC connector and the ***Arduino Case***.

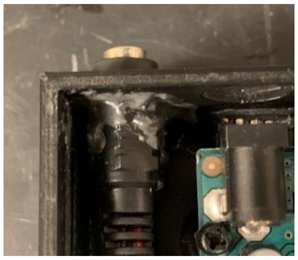

5) Leave box to dry overnight.
6) Check stability of each connector by firmly pulling on the jumper housings and DC connectors

Final Assembly

Stage assembly

Components and building materials:

- Pre-assembled components
    - ***Micropositioner***
    - ***Linear Actuator*** + ***Spout*** (single spout)
    - ***Linear Actuator*** + ***Multi-Spout Assembly*** (multi spout)
    - ***Wheel Brake***
    - ***Head-fixation plate***
    - ***Rotary Encoder***
    - ***Wheel***
    - ***Solenoid*** (1/***Spout***)
    - ***Tone Generator***
- ***Arduino Console***
- 3D printed components
    - ***Head-Fixation Stage****
    - ***Conical Mount*** *
    - ***Head-Fixation Top Piece***
    - ***Stage-to-Rotary-Encoder-Adapter****
    - ***Rotary-Encoder-to-Wheel-Adapter***
- Tools and materials
    - Aluminum breadboard (or metal plate if using magnetic mounts).
    - MF jumper wires
    - MM jumper wire or Custom MM jumper wires
    - 1x3 custom jumper housing (optional)
    - 1x4 custom jumper housing (optional)
    - optical posts (2-4x)*
    - pedestal post holders (2-4x)*
    - clamping fork (2-4x)*
    - M3 screws
    - M6 screws*
    - M6 washers*
    - Machine screws (cut to length)
    - Museum putty
    - Zip ties
    - Pointed screws that come with servo horns
    - Philips screwdriver
    - M3 Allen key
    - M6 Allen key*

** Not necessary with "3D printed stage + posts"*

<u>Instructions:</u>

1) Set the aluminum breadboard in the middle of your working area.
2) Prepare the ***Head-Fixation Stage*** (skip this step if using 3D printed stage + posts)

a. Use an M6 screw and M6 washer to attach optical posts to ***Head-Fixation Stage***. If motion is not a concern, you can get away with using the front 2 positions only.

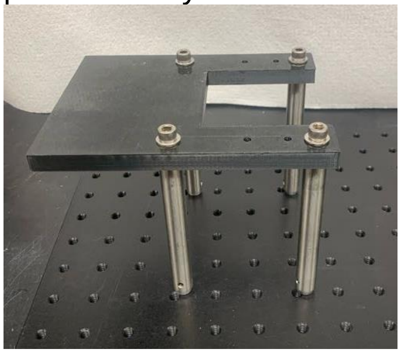

b. Attach pedestal post holders to each of the optical posts by using your fingers to tighten the locking screws.

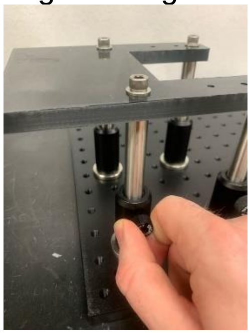

c. Stand the ***Head-Fixation Stage*** on the aluminum bread board and adjust the position like the photo below, and then loosen the locking screws of the pedestal post holders. Fix the pedestal post holders in place by placing the clamping forks over the base of the pedestal post holders and using an Allen key to drive the screw of the clamping forks into one of the threaded holes of the aluminum breadboard. Use an Allen key to tighten the locking screws of the pedestal post holders.

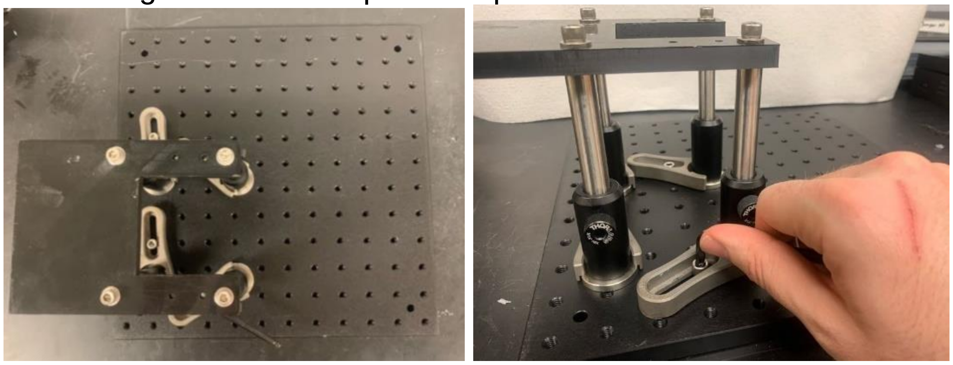

d. Attach the ***Rotary Encoder*** and ***Rotary Encoder*** to the ***Stage-to-Rotary-Encoder-Adapter***.
    i. Slide the ***Rotary Encoder*** into the ***Stage-to-Rotary-Encoder-Adapter***. The cable extending from the ***Rotary Encoder*** should be facing down and should slide into the cutout.
    ii. Secure the face of the encoder to the front of the ***Stage-to-Rotary-Encoder-Adapter*** using 3x M3 screws.

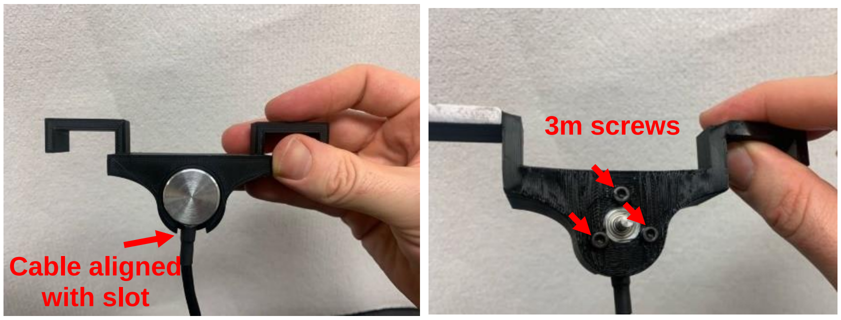

iii. Attach the ***Stage-to-Rotary-Encoder-Adapter*** to the ***Head-Fixation Stage*** placing the component in the open portion of the ***Head-Fixation Stage*** and sliding backwards as far as the component will go. Next, push the arms until the adapter is flush with the cutout in the stage.

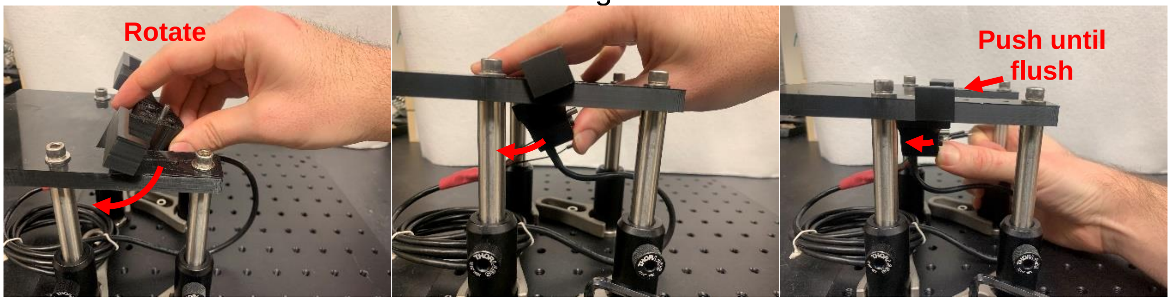

e. Attach the ***Head-Fixation Plate*** by setting it on top of the front portion of the ***Head-Fixation Stage*** with the opening of the ***Head-Fixation Plate*** facing towards the center of the stage. There are 4 holes in both pieces that should align. Insert machine screws down through the screw hole and tighten a hex nut from the bottom until secure.

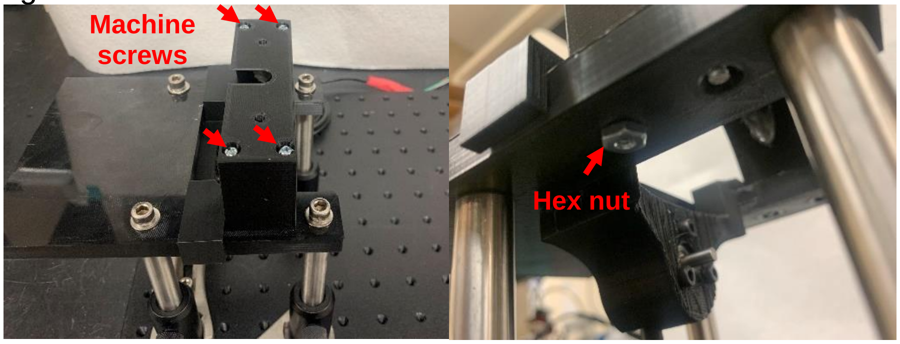

f. Attach the ***Conical Mount*** by first applying a small amount of museum putty to the rear edge of the ***Head-Fixed Stage***. Next, insert the edge of the ***Head-Fixed Stage*** into the cut out of the ***Conical Mount*** and press the ***Conical Mount*** forward until the edge has reached the end of the cut out. Firmly pinch the two parts together.

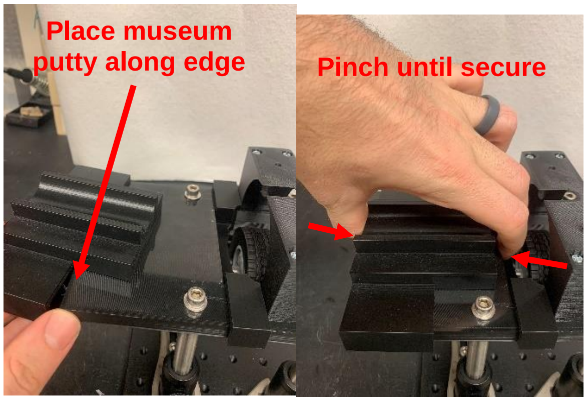

3) Attach the ***Wheel*** to the ***Rotary Encoder*** to ***Rotary-Encoder-to-Wheel-Adapter*** by carefully pressing the ***Wheel*** onto the ***Rotary-Encoder-to-Wheel-Adapter***. Next, with the ***Wheel*** attached to the ***Rotary-Encoder-to-Wheel-Adapter***, slide the adapter onto the ***Rotary Encoder*** until it cannot slide any further.

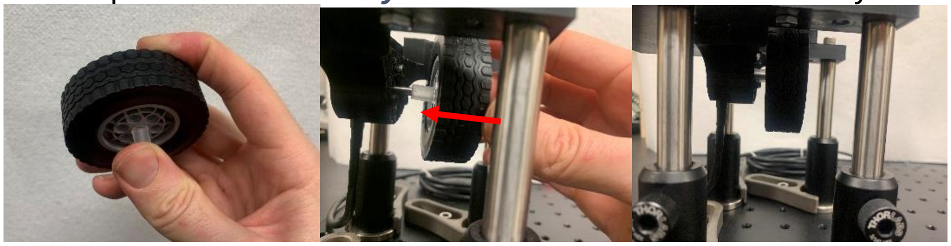

4) Fix ***Tone Generator*** to ***Stage-to-Wheel-Brake-Servo-Adapter*** and attach to ***Stage***.
    a. Secure the ***Tone Generator*** to the front of the brake mount using 2 screws (pointed ones that come with servo horns) passing through the holes in the ***Tone Generator*** and into the holes in the brake mount.

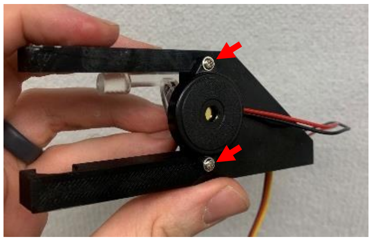

    b. Lift the ***Brake Bar*** as vertical as possible.
    c. Clip the ***Stage-to-Wheel-Brake-Servo-Adapter*** onto the posts by first connecting the side with 1 clip onto the right side of the stage (while looking towards the stage from the front), and then clip the other side with 2 clips.

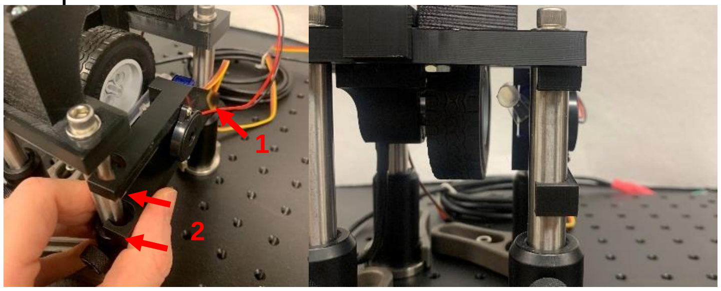

5) Set up the ***Micropositioner*** and single ***Spout*** or ***Multi-Spout Assembly***.
    a. Secure the ***Micropositioner*** base to the aluminum bread board using an M6 screw and M6 washer.

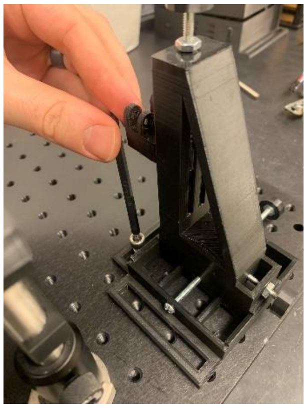

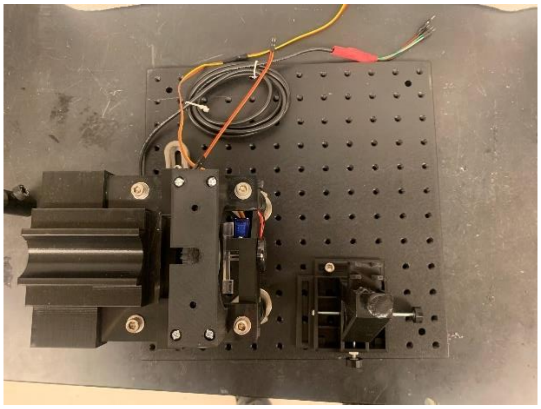

    b. Attach the ***Cover*** of the ***Linear Actuator*** to the ***Z-Adapter*** on the ***Micropositioner*** by holding a hex nut in the hex inset within the rear side of the ***Z-Adapter*** and then screwing a screw in place using a Philips screwdriver. Repeat for the second screw.

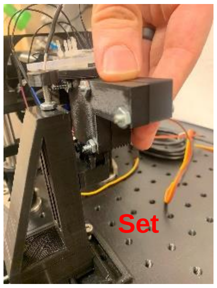

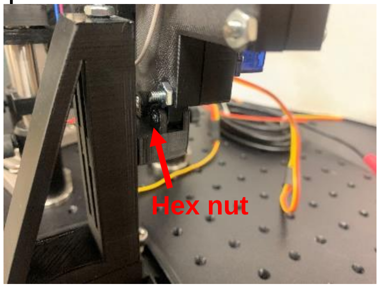

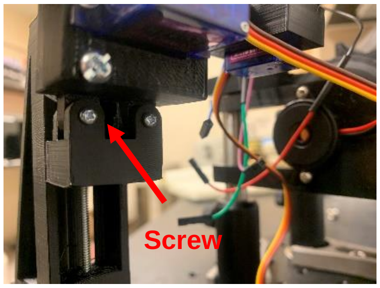

    c. Place the ***Micropositioner*** with ***Spout Assembly*** such that the ***Spout*** is directly under and perpendicular to ***the Head-Fixation Top Piece***. Remove the M6 screw holding the ***Micropositioner*** if necessary to move the ***Micropositioner*** into a good position.

6) Move ***Head-Fixation Stage*** and ***Arduino Console*** into the behavioral box adjacent to one another or fix the ***Arduino Console*** to the aluminum breadboard

Connecting electrical components

Components and building materials

- Pre-assembled components:
    - Assembled ***Head-Fixation Stage***
    - Assembled ***Arduino Console***
- Tools and materials
    - Jumper wires (MF) (*optional: custom jumper wires + housing*)
    - Wire (solid 23AWG)
    - Wire stripper
    - 5V power adapter
    - 24V power adapter
    - Zip ties
    - Labels

In this section we will hook up all the electronic components to the ***Arduino Console*** we assembled previously. The wires from the components on the ***Head-Fixation Stage*** will need to be extended by attaching additional jumper wires, custom jumper wires, or by cutting and stripping solid 23AWG wire.

Box External Connectivity Key

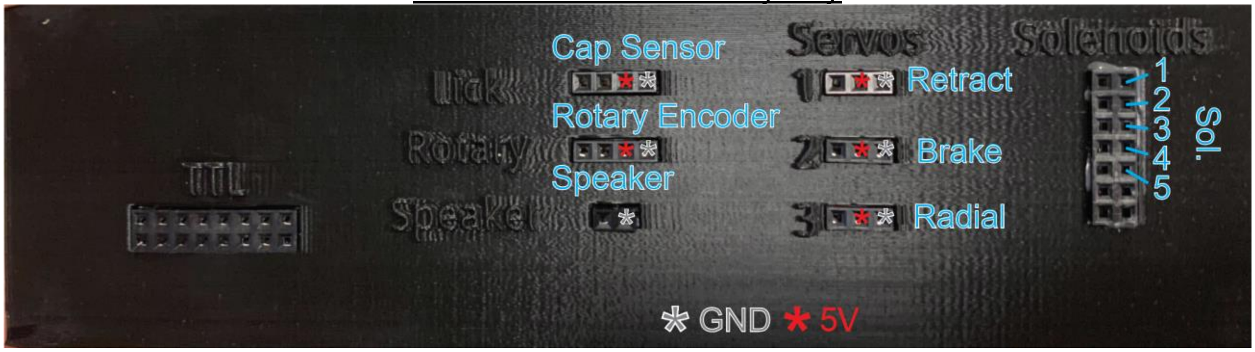

1) Lick Detection
    a. Get 4 MF jumper wires or create a custom MF jumper wire with 4 1x1 housing on the F side and a 1x4 housing on the M side.
    b. Connect the 4F pins to the VIN, GND, SCL, and SDA pins on the capacitive touch sensor.
    c. Connect the 4M pins to the row of F pins on the ***Arduino Console*** labeled "lick" (use a MF extender if the wire does not reach).
    d. Double check to ensure that the connectivity is correct. If the connections are not correct, rearrange the F pins connected to the capacitive touch sensor. The correct connectivity to the ***Arduino Console*** is:
        i. GND connected to far-right pin
        ii. VIN connected to second right pin
        iii. SCL connected to the second left pin (connected to pin 21 on the Arduino).
        iv. SDA connected to far-left pin (connected to pin 20 on the Arduino).

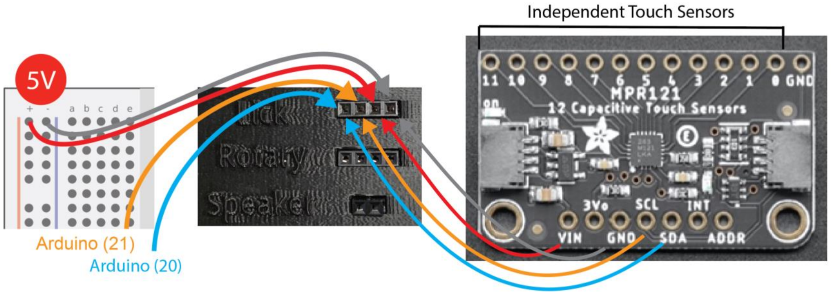

    e. Zip tie the sensor on the ***P-Body*** / ***Cover*** of the ***Linear Actuator***, or the ***Z-Frame*** of the ***Micropositioner***.

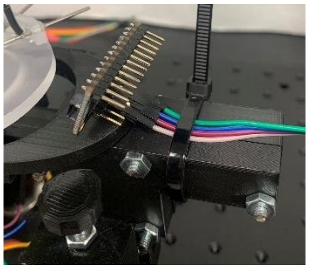

f. Connect ***Spout(s)***
   i. Single Spout: connect the F side of the alligator clip of the Lick detector clip (single ***Spout***) and clip the M side to the ***Spout***.
   ii. Multi Spout: connect the F side of each wire connected to each ***Spout*** to the capacitive touch sensor in clockwise order.

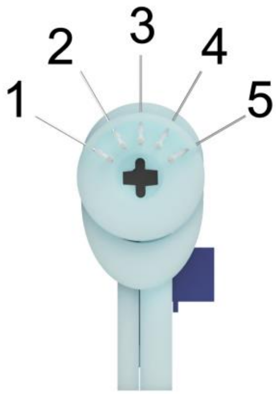

2) ***Servo***s
   a. For each ***Servo***, get 3 MF jumper wires or create a custom MM jumper wire with a 1x3 housing on each side.
   b. Attach one side to the 3 wires from the ***Servo***
   c. Attach the other side to the ***Arduino Console*** such that:
      i. GND (brown) is connected to right pin
      ii. VIN (orange) is connected to the middle pin
      iii. PWM (yellow) is connected to left pin (Arduino 9-11)

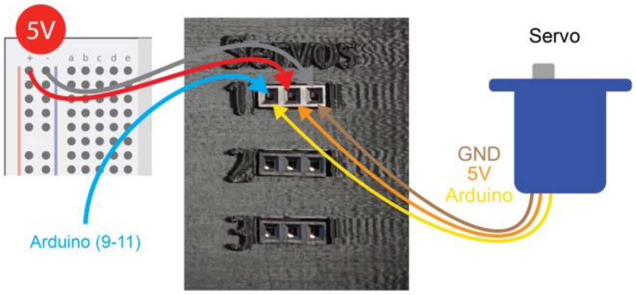

3) Tone Generator
   a. For each ***Servo***, get 2 MF jumper wires or create a custom MM jumper wire with a 1x2 housing on each side.
   b. Attach the F side to the 2 wires from the ***Tone Generator***.
   c. Attach the M side to the ***Arduino Console*** such that:
      i. GND (black) is connected to right pin
      ii. Positive (red) is connected to the left pin (Arduino pin 12)

4) ***Rotary Encoder***
   a. Get 4 MF jumper wires or create a custom MF jumper wire with a 1x4 housing on each side.
   b. Attach the F side to the 4 wires from the ***Rotary Encoder*** (order: A, B, 5V, GND, *see colors below*)
   c. Attach the M side to the ***Arduino Console*** such that:
      i. GND (white) is connected to far-right pin
      ii. VIN (brown) is connected to second right pin
      iii. B (green) is connected to second left pin (Arduino pin 3)
      iv. A (grey) is connected to far-left pin (Arduino pin 2)

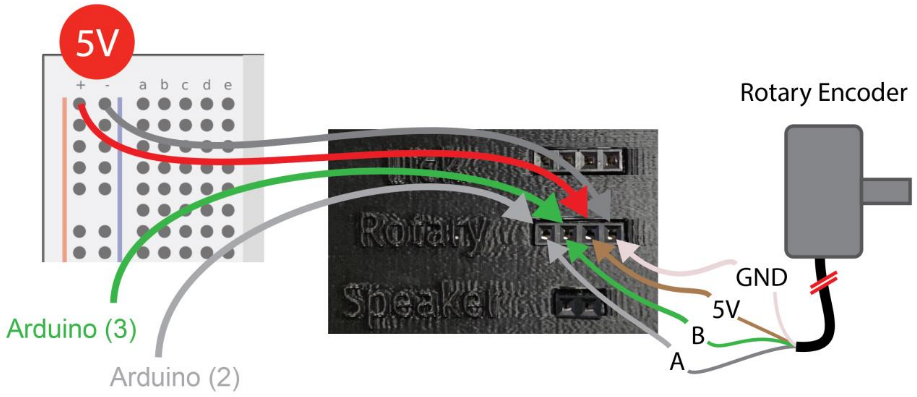

5) Power
   a. Plug the 5V and 24V power adapters into a power strip
   b. Clearly label the adapter and DC ends of each power adapter with the corresponding voltage.
   c. Plug the DC end into the corresponding DC pig tail.

Liquid delivery

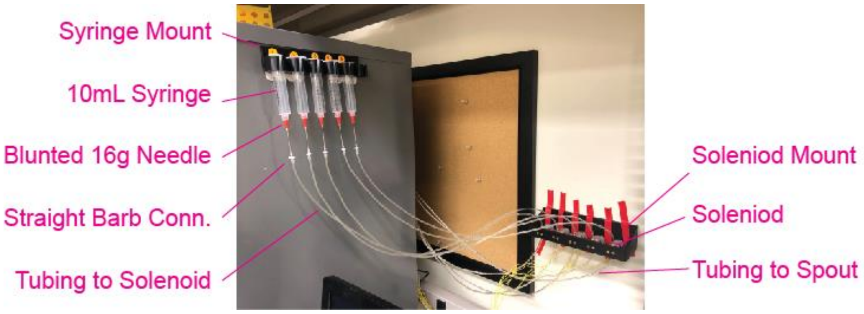

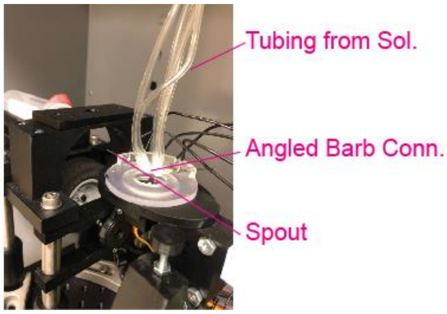

1) Fix the ***Syringe Mount*** and ***Solenoid Mount*** onto the side of the box or a nearby wall using 4x 1" command strips. Different behavioral boxes produce varying levels of noise when the ***Solenoid*** opens. If you choose to mount the ***Solenoids*** directly to the box, make sure to test the noise level produced during ***Solenoid*** operation.
2) For each ***Syringe***, dull a 16-gauge needle using a Dremel and connect it to the ***Syringe***. Next, carefully press the straight barb connector onto the 16-guage needle forcefully until secure.
3) Cut 2 sets of tubing that connects: 1) the ***Syringe*** to ***Solenoid*** and 2) the ***Solenoid*** to each ***Spout***. You will need 1 set of tubing for each ***Spout***. Make sure the length of tubing within each set is consistent. Stretch each end of the tubing so it can stably couple with a straight barb connector on one side and the ***Solenoid*** on the other side. To stretch the tubing, use a pair of hemostats or plyers and forcefully insert into the end of the plastic tubing and then open the tool until the tubing begins to go opaque. Repeat this step until the tubing on each side is wide enough to securely fit onto the corresponding connector.
4) Couple the first set of tubing to the barb connector attached to the ***Syringe*** and the barb connector on the ***Solenoid***, couple the second set of tubing to the barb connector on the ***Solenoid*** and the straight or elbow connector on the ***Spout***.
5) Attach ***Solenoid*** leads to the ***Arduino Console*** (1 row on console / ***Solenoid***).

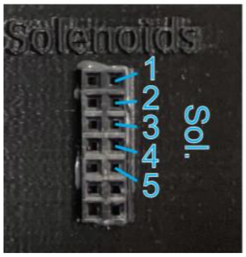

6) Check to ensure there is smooth flow through each of the lines
   a. Fill ***Syringe(s)*** with ~10mL DI water.
   b. Place the ***Spout*** into a container or hold a container directly under / in front of the ***Spout***.
   c. Open ***Solenoid(s)*** using the Arduino script *helper_opensol.ino*.
   d. Push a 10mL plunger into the ***Syringe*** and continue to depress until liquid flows freely through the ***Spout***
   e. TROUBLE SHOOTING: If you notice that an excessive amount of force is necessary for flow, then replace the straight barb connector on the

Syringe and try again. If this fix does not work, then check to see if the ***Solenoid*** or ***Spouts*** are clogged.

Hardware checks

Check to ensure that each component of the system is working properly.

1) Check that each ***Servo*** moves using *manual_servo.ino.*
   a. Servo 1: retract (Arduino pin 9).
      i. 180 degrees should be fully extended.
      ii. 120 degrees should be retracted.
   b. Servo 2: Brake (Arduino pin 10).
      i. 0 degrees should be fully lifted.
      ii. ~20-30 degrees should brake the wheel.
   c. Servo 3: Radial (Arduino pin 11).
      i. ~0 degrees should position the ***Spout*** for ***Spout*** 1.
      ii. ~150 degrees should position the ***Spout*** for ***Spout*** 5.
2) Check that each ***Solenoid*** opens using *manual_pin.ino.*
   a. ***Solenoid(s)*** located on pins 4 up to 8 should open when pin is set to high.
   b. When ***Solenoids*** open, there should be an audible click and liquid should flow through the line
   c. TROUBLE SHOOTING: if the ***Solenoid*** is not making an audible click, check to see if the ***Solenoid*** is opening silently by placing your fingers on the corresponding ***Solenoid*** while opening and closing (if the ***Solenoid*** is operational, there should be a subtle pulse each time it is opened and closed). If the ***Solenoid*** is confirmed to be not opening, check to ensure that 24V is being provided to the ***Arduino Console***. Next, check to ensure the correct pin is being controlled via *manual_pin.ino*. Finally, check the wiring of the ***Solenoid*** circuit to ensure that everything is hooked up properly. To check to ensure the ***Solenoid*** is operational, plug the ***Solenoid*** directly into the 24V + / -,the ***Solenoid*** should open the moment it is connected.
3) Check that each ***Spout*** detects licks using *MPR121test.ino.*
   a. If necessary, download the MPR121 library (installed through Tools > Manage Libraries > search MPR121).

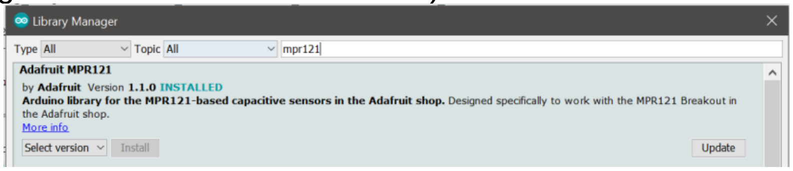

   b. Open *MPR121test.ino* (File > Examples > Adafruit MPR121 > *MPR121test.ino*) and upload to Arduino.

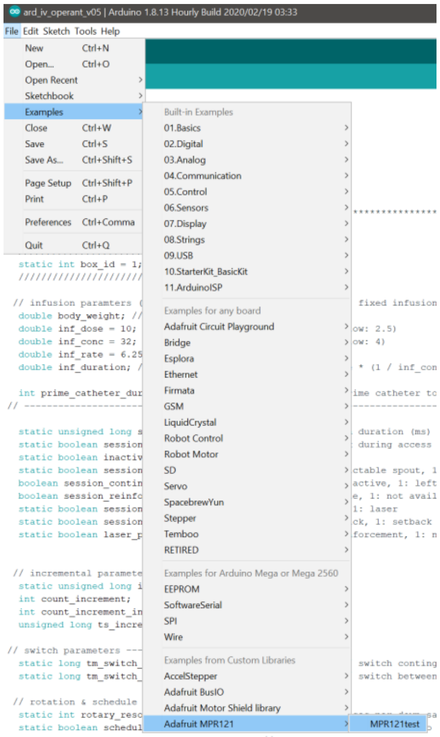

c. Open serial monitor (making sure that baud rate is set correctly), then touch each ***Spout*** individually. Each contact and release should be displayed for each ***Spout*** in the system.

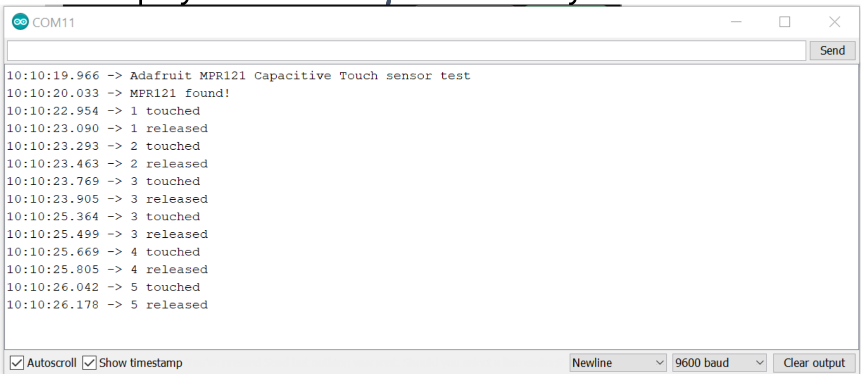

TROUBLE SHOOTING: If the MPR121 is not detected, check the connectivity, and ensure that the 5V adapter is connected.
TROUBLE SHOOTING: If the MPR121 is detected but touch is not registered, try dipping your finger into water before touching the ***Spout*** (dry skin can sometime not be detected).

d. Check the resting capacitance for each ***Spout***.
   i. Comment out line with 'return;'.

```
// comment out this line for detailed data from the sensor!
//return;
```

   ii. Re-upload script and open serial monitor.
   iii. Serial monitor will now display the live capacitance measurement for each ***Spout***.

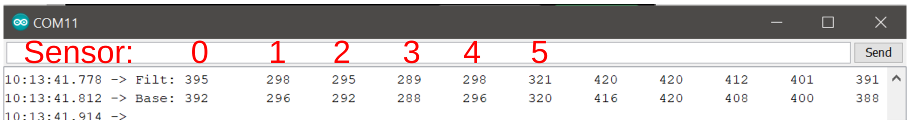

iv. The resting capacitance is a function of the amount of material connecting the ***Spout*** to the cap sensor, the size and material of the ***Spout***, and the properties of the liquid within the ***Spout***. Higher numbers here indicate lower capacitance readings. The resting capacitance should have a value greater than 120, as values lower than this will lead to poor lick detection with mouse tongue contact. TROUBLE SHOOTING: if the value is too high, try reducing the length of the wires from the capacitive touch sensor to the ***Spout(s)***. Additionally, make sure that the wires from the ***Spout(s)*** are not bundled together.

4) Check that ***Rotary Encoder*** is working using *test_rotaryencoder.ino.*
   a. Open test_rotaryencoder.ino and upload to Arduino.
   b. Open serial monitor.
   c. Once "Test for ***Rotary Encoder***…" is displayed, rotate the wheel left and right.

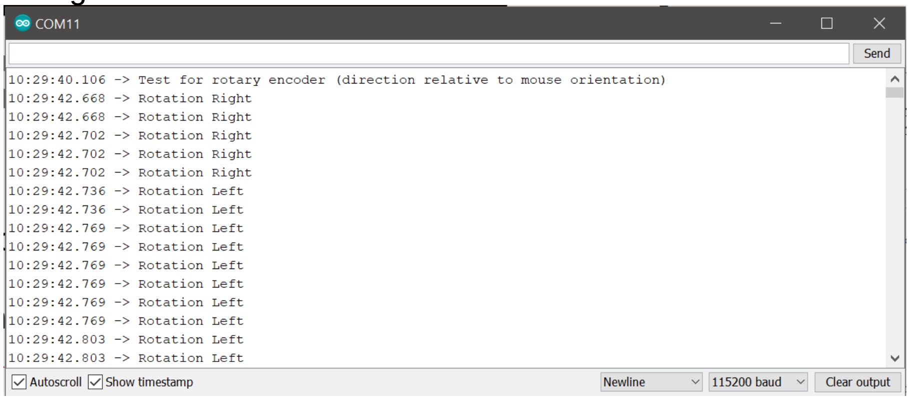

   d. Check to ensure direction is correct relative to the mouse's perspective TROUBLE SHOOTING: if direction is backwards, reverse connections for Pin A and Pin B at the connection between the ***Rotary Encoder*** and ***Arduino Console***.
   TROUBLE SHOOTING: If no rotation is being detected, check the connectivity, and ensure that the 5V adapter is connected.

Cable management / tidying

Once all hardware is tested and confirmed to be working:

1. Cover the ***Arduino Console*** using the 3D printed cover (*option: glue magnets into the cube insets in the **Arduino Console** and Arduino cover for a magnetic lid*).
2. Coil up free jumper wires and zip tie to tidy up stage.

Next steps

Before running behavior procedures, you may need to calibrate hardware components. Refer to each behavioral program's specific protocol for additional calibration steps.
